# Graphic health warnings and plain packaging in the Philippines: results of online and household surveys

**DOI:** 10.3389/fpubh.2023.1207779

**Published:** 2023-09-26

**Authors:** Gianna Gayle H. Amul, Eunice U. Mallari, John Rafael Y. Arda, Alen Josef A. Santiago

**Affiliations:** Ateneo Policy Center, School of Government, Ateneo de Manila University, Quezon City, Philippines

**Keywords:** addiction, low/middle income country, packaging and labeling, public opinion, advertising and promotion

## Abstract

In line with Article 11 of the WHO Framework Convention on Tobacco Control, the Philippines has implemented graphic health warnings on cigarette packs. To date, there has been no published literature evaluating the perceived effectiveness of GHWs in the Philippines. This study aims to contribute to the evidence on the perceived effects of graphic health warnings (GHWs) in cigarette packaging and the potential impact of plain packaging in the Philippines. The study involved an online convenience survey and a nationwide household survey. Mock-up sets of cigarette packs based on the Philippines’ law on GHWs, and Thailand’s and Singapore’s plain packaging were shown to respondents to rate their attractiveness, quality, taste, cost, social appeal, appeal to youth, noticeability, appeal to non-smokers, attempt to quit, ease of quitting, discouraging smoking, and perceived harm to health on a five-point Lickert scale. The online and household surveys recruited 2,701 respondents in total. Online and household survey respondents considered plain packaging with larger graphic health warnings and visible quitlines from Thailand and Singapore to be more effective in discouraging them from smoking. Both sets of survey respondents also found mock-ups from Thailand and Singapore more motivating for them to attempt quitting than cigarette pack mock-ups from the Philippines. The study concludes that current graphic health warnings in the Philippines are ineffective in instilling health consciousness among Filipinos. Policymakers should consider larger graphic health warnings and plain packaging of cigarettes to motivate smokers to quit and discourage Filipinos from smoking.

## 1. Introduction

The Philippines’ smoking prevalence remains high despite implementing demand, supply, and harm reduction tobacco control measures since the early 2000s. According to the 2015 Global Adult Tobacco Survey (GATS), 23.8% of the adult population uses tobacco, with the majority being smokers (22.7%) (1). Eight of the top 10 leading causes of death are associated with smoking tobacco (2). The country has a high burden of smoking-attributable non-communicable diseases (NCDs) and would benefit from tobacco control policies.

In 2003, the Philippines became a signatory to the World Health Organization Framework Convention on Tobacco Control (WHO FCTC), the first evidence-based international health treaty negotiated under the WHO (3). The WHO FCTC includes demand, supply, and harm reduction measures (3). The Philippine Senate ratified the WHO FCTC in 2005, which effectively validated the WHO FCTC as part of the national law (4). As a party to the WHO FCTC, the country agreed to adopt non-price measures to reduce tobacco demand through packaging and labeling measures (3). The Philippines’ Graphic Health Warnings (GHWs) Law was drafted and implemented with consideration to the Philippines’ obligations to the WHO FCTC “to inform every person of the health consequences of tobacco use and exposure” (5). The law also referred to the empirical evidence that text warnings on cigarette packs were insufficient and that graphic health warnings were more effective in informing the public of the health harms of exposure to tobacco smoke and tobacco use (5).

Per the Philippines’ Republic Act (RA) No. 10643, otherwise known as the Graphic Health Warnings Law, GHWs are “photographic images printed on the tobacco product package which accurately depict the hazards of tobacco use and are accompanied by textual warning related to the picture (5).” Signed in 2014 and implemented in 2016, the law introduced current GHWs on tobacco products, changing designs every 2 years (5). These are printed on 50% of the principal display surfaces of tobacco packages, both on the bottom side of the front and back panels and may include 20% of the space dedicated to textual warnings related to the pictures. Additional textual warnings, as well as tobacco-related quitlines and websites managed by the Department of Health (DOH), may occupy up to 30% of the space on the side panels (6). Compared to its Association of Southeast Asian Nations (ASEAN) neighbors, the Philippines is lagging in implementation. It is the only country that does not require health warnings on the top front and back panels of the package (6).

To date, systematic reviews from high-income and middle-income countries suggest that larger GHWs are associated with increased salience and knowledge on smoking harms and health risks and that larger GHWs are more effective in encouraging behavioral change, particularly in influencing intentions to smoke (foregoing cigarettes) and intentions to quit smoking (e.g., quitline calls, quit attempts, and short term cessation) and decreasing smoking prevalence (7–11). There is also increased effectiveness when larger and new GHWs are introduced in regular intervals to sustain salience (8, 9). Recently, a global ecological time-series analysis of the comprehensive and simultaneous implementation of smoking bans, GHWs, advertising bans, and tobacco taxes confirmed tobacco control measures’ effectiveness in reducing smoking prevalence (12). The international evidence on plain packaging highlight how plain packs increase the noticeability and impact of GHWs (12), avoid false and misleading descriptors about cigarettes (13–15), and reduce positive impact including the appeal of smoking to young people (16–18).

To better guide the implementation of tobacco control measures, this research seeks to provide local evidence on the effects of Philippine-designed GHWs and the potential impacts of plain packaging in the Philippines. This will include the perception of the participants on different GHWs and potential plain packaging. Specifically, the study aims to assess: (1) the efficacy of the Philippines’ current round of GHWs in (a) instilling health consciousness, (b) educating about the harms of smoking, and (c) encouraging smokers to quit, and (2) assess the potential and acceptability of plain cigarette packaging and larger GHWs in the Philippines.

## 2. Materials and methods

This study is part of the project Strengthening Pack Warnings on Tobacco Products in the Philippines, which aims to provide empirical evidence on the effect of GHWs on reducing cigarette demand and other health outcomes, and the potential impacts of plain packaging in the Philippines, by the Ateneo Policy Center (APC) under the Ateneo School of Government (ASoG), Ateneo De Manila University (ADMU).

### 2.1. Design

**Figure 1:**
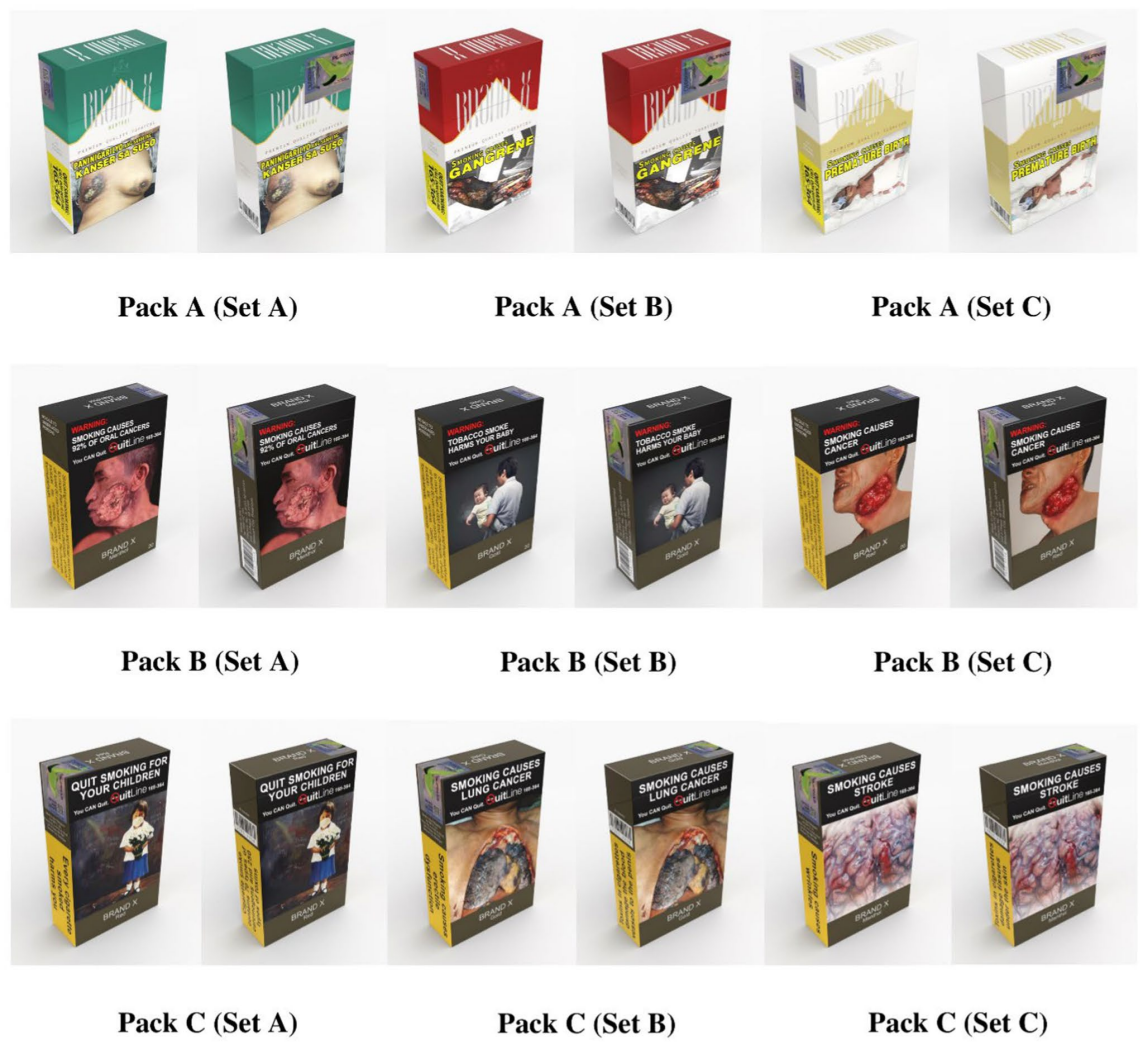
Pack designs for each set based on the Philippines’, Singapore’s, and Thailand’s current GHWs, respectively.

The study is a result of both online and household surveys. We designed and developed a survey questionnaire to gather data on smoking status, intention to quit, GHW perceptions, and the potential impact of plain packaging in the Philippines. The first section asked respondents questions about their demographic profile, smoking status, intention to quit, use of e-cigarettes, and exposure to cigarette packs with GHWs and plain packaging. Three cigarette packs were shown to respondents: (1) the Philippines’ current GHWs focused on health threats; (2) Singapore’s enlarged (85% of the pack) GHWs (front and back) focused on health and social threats, and (3) Thailand’s enlarged (85% of the pack) GHWs (front and back) focused on health and social threats. Respondents were asked to assess the cigarette packs on a five-point Likert Scale (5—Strongly Agree to 1—Strongly Disagree) and according to attractiveness, quality, taste, cost, social appeal, appeal to youth, noticeability of the GHWs, appeal to non-smokers, attempt to quit, ease of quitting, discourage to smoke, and perceived harm to health. Respondents were also asked for additional comments about each cigarette pack shown. At the end of the questionnaire, respondents determined which they think is most effective in convincing them not to smoke among the three packs shown. Figure 1 shows the set of packs used in both the online and household survey. See Supplementary Material 1 for a copy of the survey instrument.

The survey design is based on Singapore’s Health Promotion Board’s local survey in 2015 on current cigarette packaging and (mock-up) plain cigarette packaging adapted to the Philippine context. Several questions on demographics and exposure to GHWs were adapted from the GATS. Questions on e-cigarette use were included in consultation with the Philippines’ DOH.

### 2.2. Pilot testing of the survey questionnaire

A pilot run of the survey was conducted through an online focus group discussion (FGD) and an online survey via Google Forms. We had five participants that joined the FGD via Zoom and we recruited them via the personal network of the researchers. They were a combination of two smokers, one non-smoker, and three occasional or previous smokers. A similar set of three packs from the survey questionnaire (See Supplementary Material 1) were shown to the participants to rate their design, GHWs, appeal, perceived ease of quitting or discouraging smoking, and perceived harm to health. The pilot online survey on Google Forms ran for 2 weeks and garnered 93 responses. Results and feedback from the FGD and pilot online survey were used to refine or improve the survey questions. Comments on the survey included adding Occasional Smoker as a smoking profile, using a five-point Likert Scale instead, adding a “Not Applicable” option for non-smokers or to some specific questions, and clarification on the options to answer the age question. See Supplementary Table 1 for the demographic profile and results of the pilot online survey.

### 2.3. Data collection procedure

#### 2.3.1. Online survey administration

The research team used ADMU’s Google Suite to deploy a self-administered online survey on Google forms as a secure platform for data collection. Three different packs were deployed as shown in Figure 1. The link to the survey was posted with the advertisement via the ASoG Facebook account, and it openly invites followers of the page to answer the survey. See Supplementary Material 2 for the advertisement that was posted. The ASoG Facebook page has 15,000 Likes and 16,000 Followers as of this writing. Most of its followers are researchers and people from academe and government institutions. The Facebook page mainly posts Events, Invitations, and News about the School of Government. We boosted the post with a paid and targeted Facebook advertisement to capture the geographical representation of the respondents (18–65 years old) and opened the advertisement to non-followers of the page. Since Meta, the company which owns Facebook, acquired Instagram, advertisements on Facebook are also shown on Instagram. Target respondents who clicked the link to the survey instrument were then randomly directed to a particular set of Google Forms through a link to a random Google Form generator coded through Google Script. Randomization ensured an appropriate number of responses for each set of packs. The landing page of the Google Form provided objectives of the study, what it entails to participate, name and contact information of the research team, and the national quitline for respondents who are smokers to contact to start a smoking cessation program. Respondents can also access the informed consent form on the landing page of the Google Form. Clicking on “proceed to the survey” button means consent to participate, and participants were directed to the questionnaire. The Facebook advertisement ran for the whole month of March 2021 (31 days) until the quota (*n* = 500) for each set was met for a total target sample size of 1,500. The study required an email address to be collected to authenticate respondents, avoid spamming, and limit respondents to one response only. The email addresses were permanently deleted once the survey period ended. No personal data were extracted. Responses that were beyond the target sample for 18–65 years old residing in the Philippines were removed from the final count of survey responses. As of this writing, the Facebook advertisement garnered an estimated 4,600 likes, 604 comments, and 324 shares. The targeted Facebook advertisement of the survey reached an estimated 266,000 Facebook and Instagram users in the Philippines.

#### 2.3.2. Recruitment of data collectors for the household survey

The study recruited local data collectors as gatekeepers to the study communities and to facilitate the data collection of the household survey. We recruited local data collectors based on their familiarity with the local language, local community, proximity or residency in the local community, and experience in data collection for household surveys. This was not only to ensure familiarity with the local community and comprehension of participants of the survey question but also to minimize the transmission of COVID-19 to both data collectors and selected areas and communities. The research team conducted a virtual data collection training and briefing (over Zoom) to familiarize them with the survey instrument, as well as the necessary health, safety, and ethical research protocols. We also recruited four regional field data supervisors to supervise and guide the data collection process. This ensured the timely completion of our survey as well as the ethical and safe implementation of the household survey.

#### 2.3.3. Household survey administration

Despite restrictions brought about by the COVID-19 pandemic, the research team also conducted a household survey to capture segments of the Philippine population more vulnerable to smoking that cannot be captured by the online survey. The household survey recruited a sample size of 1,200 representatives from the target population of 18–65 years old residing in the Philippines. The sample size falls within the range of other accepted national surveys such as those conducted by the Social Weather Stations (SWS) and Pulse Asia Research Inc., two major public-opinion polling bodies in the Philippines. Through a multi-stage stratified sampling approach, we randomly selected five cities or municipalities from each regional strata [Metro Manila or National Capital Region (NCR), Luzon, Visayas and Mindanao] to identify the enumeration area and to divide the population sample. We generated a total of 300 representative samples coming from three barangays (the basic political unit in the Philippines) of selected cities or municipalities of regional strata. Data collectors identified a starting point or a landmark in each barangay as a random start and subsequently, data collectors observed an interval sampling of every fifth household. We invited only one eligible adult in the household to participate.

The household survey was implemented from March to July 2021. The household survey in Metro Manila or NCR was delayed because NCR is the epicenter of the COVID-19 surge. This timeline included the postponement of the data collection activities because of the sudden local surges of COVID-19 cases and other circumstances encountered during the data collection. The local data collectors contacted the randomly selected household with a maximum of three attempts. They explained the purpose of the study, salient parts of the informed consent forms (ICF), and the survey methods. Local data collectors were reminded to follow standard health protocols such as physical distancing measures and the use of level 2 personal protective equipment (PPE). When an eligible respondent of a household agreed to participate, the local data collectors discussed the options to answer the self-administered survey questionnaire: (a) data collectors leave the survey packet in the household and will return to the household for the accomplished survey, or (b) the data collectors stay while the respondent answers the questionnaire upon distribution and data collectors can respond to any questions regarding the survey. Most of the respondents preferred the second option.

### 2.4. Data analysis: quantitative and qualitative approaches

We used descriptive statistics including frequencies, means, and percentages to summarize the demographics and smoking status of the participants. Furthermore, we separately reported the analysis for smokers and non-smokers to account for smokers’ preconceptions of current cigarette packs. We used Spearman rank correlations to test for associations between attractiveness and packing attributes among the three packaging designs. All data were analyzed using STATA 15. The level of significance was set at *p* < 0.05.

### 2.5. Patient and public involvement

Patients or the public were not involved in the design, conduct, reporting, or dissemination plans of our research.

## 3. Results

### 3.1. Demographic profile and smoking status

**Figure 2:**
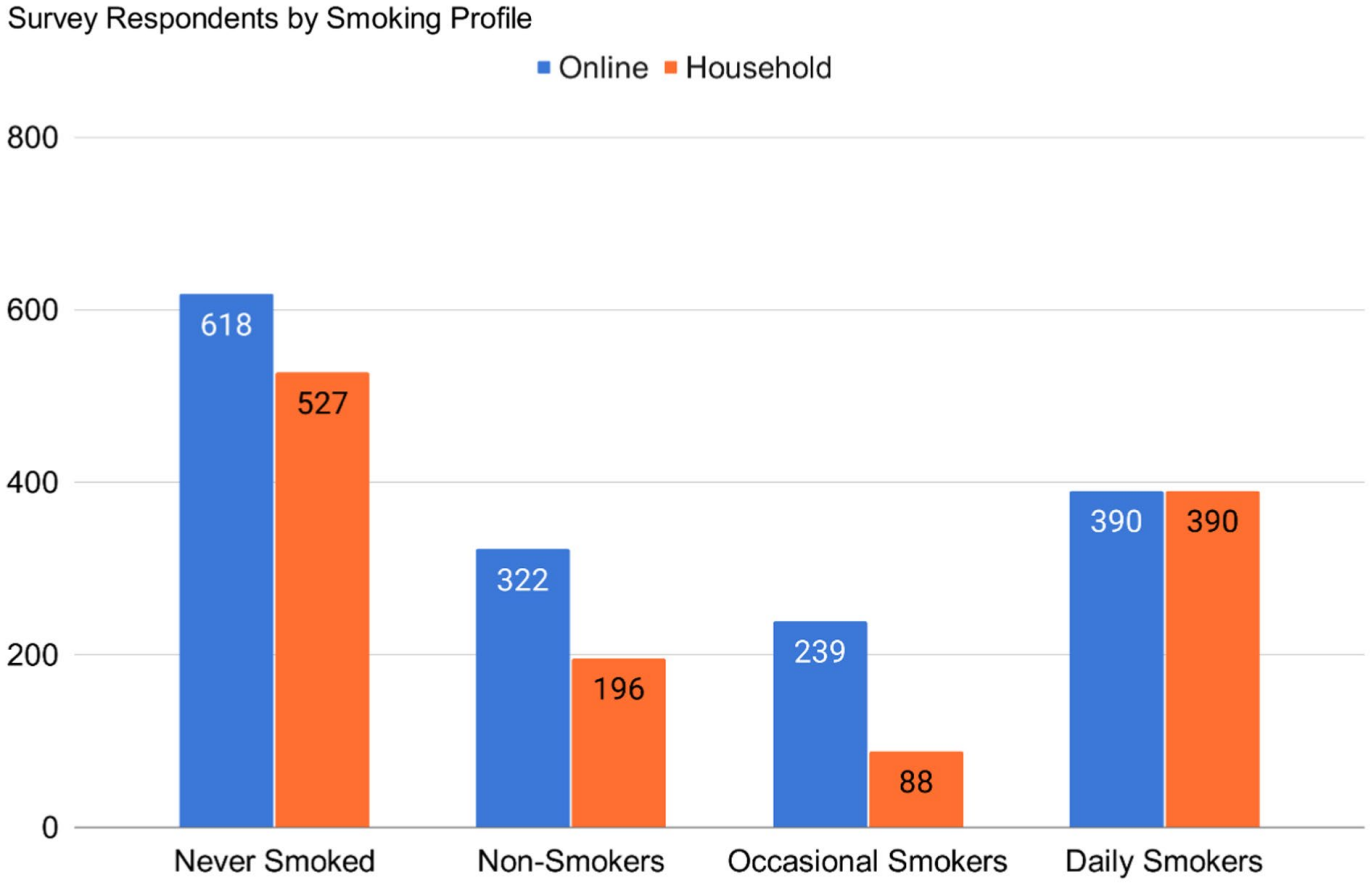
Smoking profile distribution for online and household survey respondents.

We recruited a total of 2,701 respondents. This comprised 1,500 respondents and 1,201 respondents who took the online (March 2021) and household survey (from March to July 2021), respectively. Supplementary Tables 2, 3 show the socio-demographic characteristics of each, respectively. We classified each respondent according to four smoking profiles. Both survey types returned similar smoking profile distributions, with 41.2% in the online survey and 43.9% in the household survey responding to having never smoked and classified as never smokers. Figure 2 illustrates this for both survey types.

Reflecting the nature of online social media-based recruitment, the majority of the online survey respondents regardless of smoking status came from NCR (42.5%, *n* = 638) and Region IV-A (20.8%, *n* = 312), and most of the respondents were aged 18–24 (59%, *n* = 885), were students (42.3%, *n* = 634), mostly college graduates and undergraduates. Among the online survey respondents, 37.3% (*n* = 560) were smokers (daily and occasional), 21.5% (*n* = 322) were previous smokers, and 41.2% (*n* = 618) were never smokers.

Despite COVID-19 restrictions being implemented during the period of the household survey, the data collectors were able to collect survey data from 300 household respondents from NCR, 304 respondents from Luzon, 300 respondents from the Visayas, and 297 respondents from Mindanao. Most of the household respondents were aged 35–54 (*n* = 563), graduated from high school (*n* = 383), and were self-employed (*n* = 360), Among the household survey respondents, 40% were smokers (daily and occasional smokers).

### 3.2. Smoking status, smoking behavior, and quit attempts

Of the daily smokers (*n* = 321) who responded to the online survey, 78.8% buy cigarettes by the pack, and 21.2% still buy cigarettes by the stick. On the other hand, among occasional smokers in the online survey, 54% buy cigarettes by the pack, and only 46% buy cigarettes by the stick.

Because of the geographic reach, socio-economic variation, and more representative sample for the household survey, only 19.7% buy cigarettes by the pack, and 80.3% still buy cigarettes by the stick among daily smokers who responded to the household survey (*n* = 390). On the other hand, among occasional smokers (*n* = 88) who responded to the household survey, only 10.2% buy cigarettes by the pack, and 89.8% still buy cigarettes by the stick.

For both surveys, Table 1 shows the quitting attempt among daily smokers and occasional smokers between the household and online survey respondents. This reflects the difficulty among daily smokers and occasional smokers to quit smoking.

**Table 1:** Quitting attempts by daily smokers and occasional smokers.

Quitting attempts	Household survey		Online survey	
	Daily smokers (*n* = 390; %)	Occasional smokers (*n* = 88; %)	Daily smokers (*n* = 321; %)	Occasional smokers (*n* = 239; %)
a. I have thought about quitting but not seriously and have not cut down or tried to	21.1	10.2	21.8	13.4
b. I have thought seriously about wanting to quit in the next 6 months, but I have not done anything yet	6.9	9.1	10.9	0.8
c. I intend to quit in the next 6 months and taking the steps to do so, I am currently in the process of quitting/cutting down	11.6	25.0	13.4	33.9
d. I have tried quitting but keep starting again	46.3	46.6	44.2	28.9
e. I have not thought of quitting at all	14.1	9.1	9.7	23.0
Total	100.0	100.0	100.0	100.0

### 3.3. Exposure to real-world graphic health warnings and thoughts of quitting

Among the online survey respondents, 74.5% noticed or were exposed to any health warnings on cigarette packages. Among the online survey respondents who were exposed to GHWs in the last 30 days, only 24.3% said that the GHWs made them think about quitting smoking, while 53% said that the GHWs did not make them consider quitting smoking.

The majority (89.1%) of the household survey respondents were exposed to health warnings on cigarette packages in the last 30 days. Among the household survey respondents who were exposed to GHWs in the last 30 days, 65.5% said that the GHWs made them think about quitting smoking, more than the respondents from the online survey.

### 3.4. Perceptions of respondents on mock-ups of cigarette packaging

**Figure 3:**
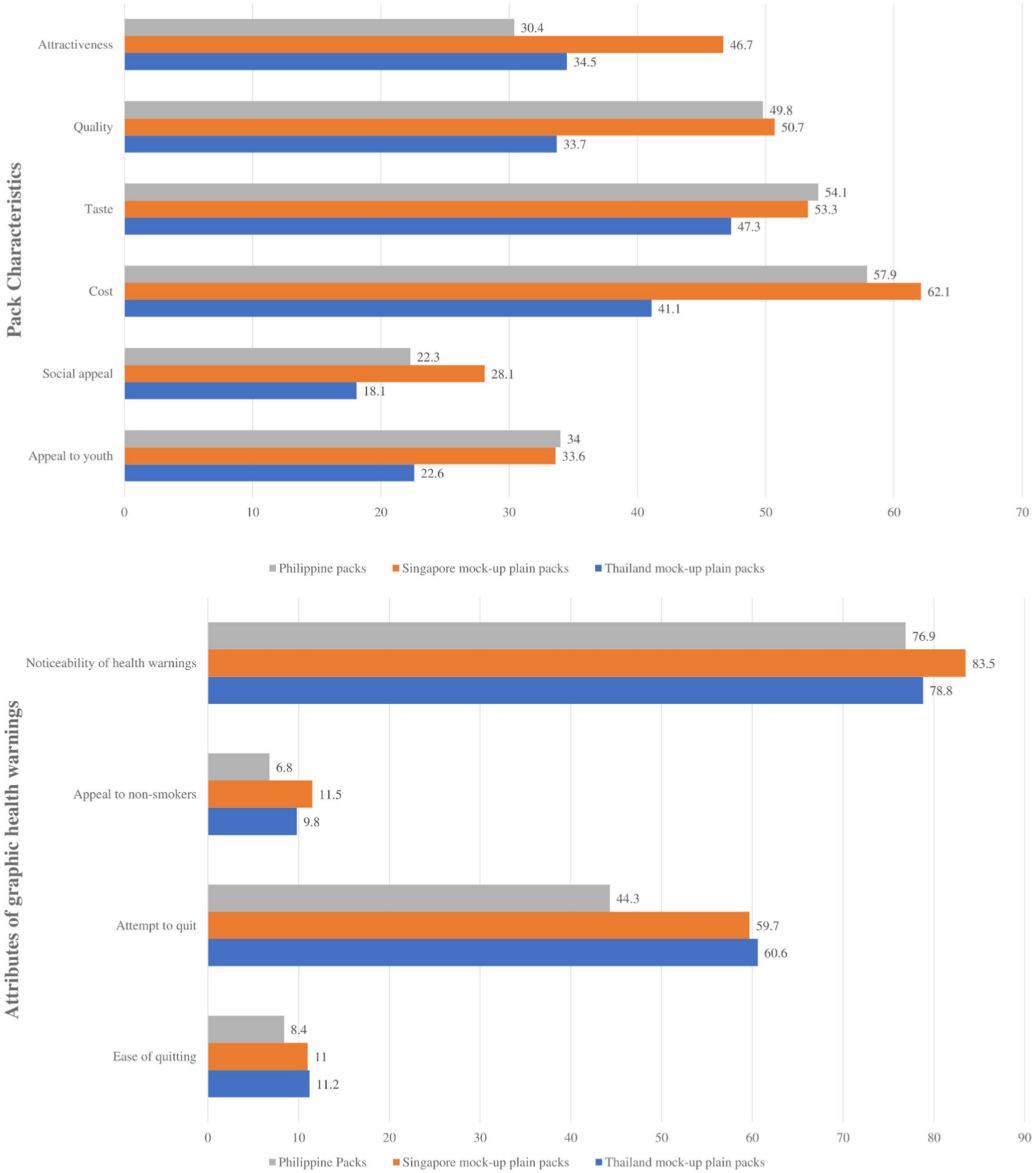
Perceptions toward pack characteristics and graphic health warnings of the current and mock-up packaging from the online survey, the Philippines, 2021.

For both online and household survey respondents, the mock-up packs from Singapore had the most noticeable GHWs but both the mock-up packs from Singapore and Thailand were more motivating for the respondents to attempt to quit than those from the Philippines. The overall perceptions of the pack characteristics and GHWs from the online survey are presented in Figure 3, while the overall perceptions of respondents on the pack characteristics and GHWs regarding smoking status are presented in Supplementary Table 4.

**Figure 4:**
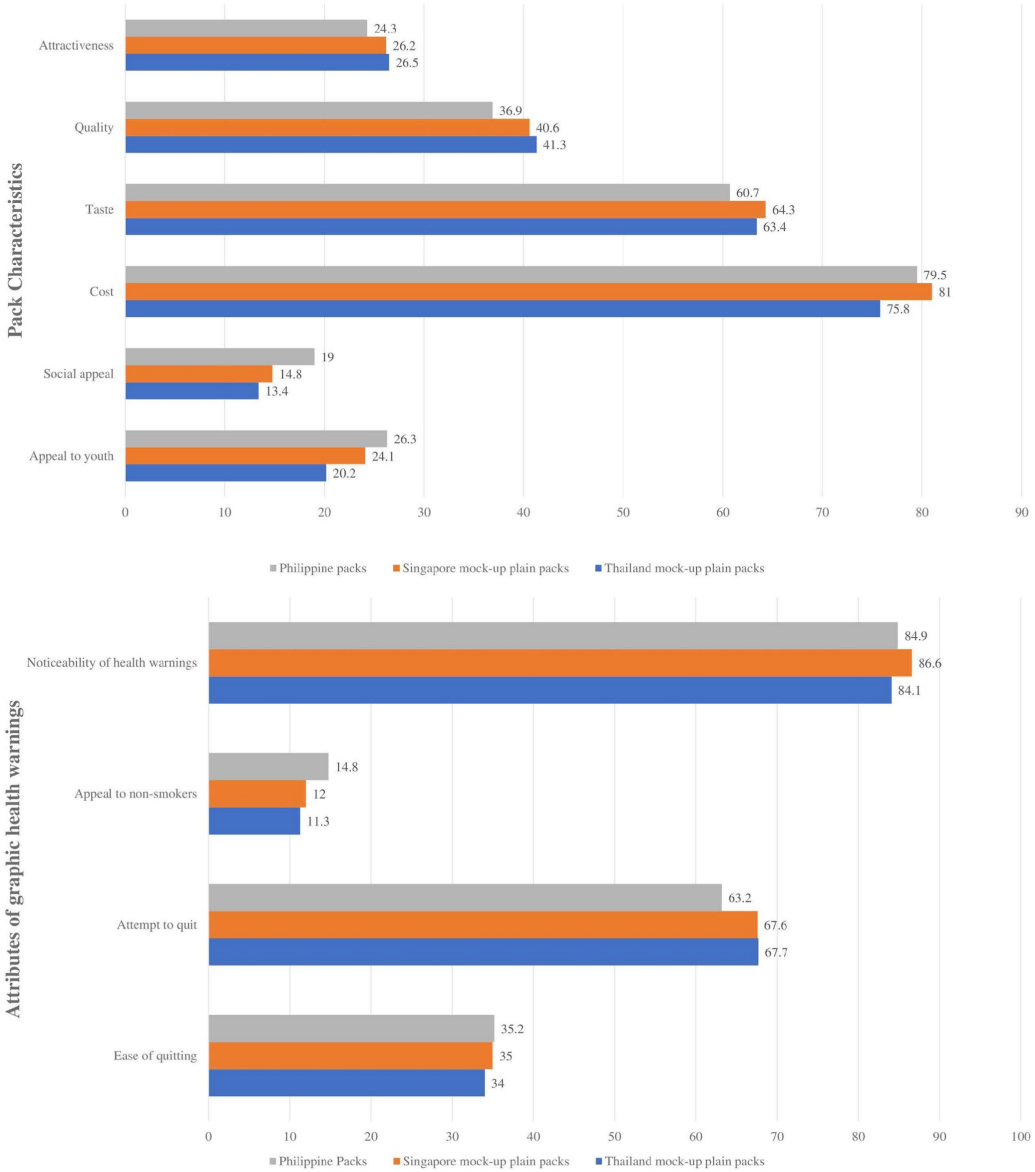
Perceptions toward pack characteristics and graphic health warnings of the current and mock-up packaging from the household survey, the Philippines, 2021.

Similarly, the overall perceptions of the pack characteristics and GHWs from the household survey are presented in Figure 4, while the overall perceptions of respondents on the pack characteristics and GHWs by smoking status are presented in Supplementary Table 5.

Pack characteristics do not seem to be an indicator of how well the attributes of a GHW work, with most of the respondents in both surveys returning varying results for the mock-ups. Among the online respondents, plain packs from Thailand were considered to be the least aesthetically pleasing while the packs from Singapore appear to be the most aesthetically pleasing (attractive and pleasing to the eye). The household respondents have mostly preferred Singapore’s plain packs as well overall, with the Philippine pack being particularly socially appealing (respondents do not mind being seen with the pack in public) and appealing to the youth (respondents consider the pack to be attractive and pleasing to the eye for the youth) while being the least desirable in terms of taste, quality, and attractiveness.

While being the most aesthetically pleasing, the Singaporean packs also had the most noticeable health warnings across both surveys and returned high results for respondents to attempt to quit smoking. Packs from Thailand had the highest results in making respondents consider and attempt quitting smoking, while being second and third in noticeability for the online and household survey, respectively. Online respondents thought it was easier to quit with Singapore and Thailand’s plain packs despite being more appealing to non-smokers, while the household respondents thought the same for the Philippines’ packs. These are reflected in the associations measured for the different attributes of the packs to its overall attractiveness. See “Associations between the attractiveness and packing attributes among the three packaging designs.”

### 3.5. Comparing current Philippine cigarette packs and mock-ups of plain packaging from Singapore and Thailand

Among the online respondents, 93.6% considered plain packaging more effective in discouraging respondents to smoke—48.5% considered plain pack cigarette mock-ups from Thailand to be the most effective; 45.1% picked the Singapore mock-ups, while only 6.4% answered current Philippine packs.

Similarly, among the household respondents, 86.1% considered the plain packaging mock-ups to be more effective in discouraging respondents to smoke—52.1% considered plain pack cigarette mock-ups from Thailand to be the most effective; 34% picked the Singapore mock-ups, while only 13.9% chose current Philippine packs.

Although responses to questions on the attributes of packs and GHWs gave mixed results, the plain packaging mock-ups were overwhelmingly seen as the most effective in discouraging respondents from smoking. The online respondents preferred Singapore’s, while the household respondents preferred Thailand’s, indicating their perceived effectiveness of plain packaging. This suggests that while the plain packaging mock-ups are not devoid of attributes seen as marketing of tobacco products, the overall idea of plain packaging itself is seen as effective across all smoking profiles in both surveys.

### 3.6. Associations between the attractiveness and packing attributes among the three packaging designs

In the online survey, all attributes of the Philippine mock-ups are associated with the perception of the attractiveness of the pack except for the noticeability of health warnings (*r* = 0.09) and the attempt to quit (*r* = 0.03). For the online responses, all attributes of the mock-up of the Singapore pack are significantly associated with the attractiveness of the pack except for the noticeability of health warnings (*r* = −0.03) and attempt to quit (*r* = 0.02). In terms of the perception of mock-up plain packs based on Thailand regulations, there is a positive moderate association between the perception of online respondents on the attractiveness of the quality (*r* = 0.42) and the social appeal (*r* = 0.40) of the cigarettes.

Likewise, in the household survey, all attributes of the design of the mock-up of the current Philippine cigarette packs are associated with the perception of the attractiveness of the pack, except for taste (*r* = 0.17, *p* < 0.05) and cost (*r* = −0.03, *p* < 0.05). Among household responses, all the pack design attributes of Singapore and Thailand mock-ups are significantly associated with the attractiveness of the pack design except for taste (*r* = 0.17 and *r* = 0.01, respectively) and cost (*r* = 0.03 and *r* = −0.07, respectively). In terms of the Philippines’ GHWs for household respondents, the attractiveness of the pack was associated with the appeal to non-smokers but not to noticeability of health warnings (*r* = 0.01), encouraging them to quit (*r* = −0.08), or ease of quitting (*r* = 0.13). In terms of Singapore’s GHWs for household respondents, the appeal to non-smokers (*r* = 0.42) and ease of quitting (*r* = 0.21) were associated with the attractiveness of the pack. When considering Thailand’s GHWs for household respondents, only the GHW’s appeal to non-smokers (*r* = 0.40) was associated with the attractiveness of the pack design.

## 4. Discussion

This study contributes to the local evidence on the effects of GHWs in cigarette packaging and the potential impact of plain packaging in the Philippines. Moreover, it adds to the international evidence that exposure to GHWs can increase health consciousness or in this case, awareness of the harms of smoking (8, 10, 19).

While our study cannot validate the behavior of the respondents in terms of their motivation and quitting attempts after the survey, previous systematic reviews of longitudinal studies have shown that GHWs increase perceived harms and quit intentions (11). Graphic health warnings, plain packaging and visual display bans have been classified as “nudges” based on a behavioral science theory based on “libertarian paternalism” (20). Policies like graphic health warnings are considered as nudges when such policies “steer citizens toward making positive decisions as individuals and for society while preserving individual choice” while exploiting cognitive biases (or patterns of human irrationality) (21). Moreover, a meta-analysis of experimental studies has also found that when assessed based on a message impact framework derived from communication, psychological and social psychological theories, graphic health warnings were more effective in increasing intentions to not start smoking and to quit smoking (22).

Notable differences between the online (convenience sample) and household (more representative sample) survey respondents have been identified, particularly in their demographic characteristics and smoking behavior. Most of the online respondents were undergraduate students or college students aged 18–24 years old, while most of the household respondents were self-employed highschool graduates around the age of 35–54 years old. Online respondents preferred buying their cigarettes by the pack, while household respondents usually bought them by the stick. But despite these differences, it is important to note the similarities in their results’ implications, even with variance in quantitative magnitude. An interesting difference to consider between the two survey populations is the high incidence of buying by the stick and GHW retention among household respondents. While buying by the stick should impact the overall exposure to GHWs, the proportion of responses point to the exposure being exclusive to buying behavior. Considering the higher incidence of thoughts about quitting among the same group, GHWs seem to be effective for the household respondents. However, there are more opportunities to influence smoking behavior in this group due to their smoking behavior in the form of cigarette stick health warnings, which have been found to be effective amidst the loss of effectiveness over time of GHWs (19). Leading countries in tobacco control, such as Canada, the United Kingdom, and Australia, are considering its implementation (22–24).

Evidence from the study can aid legislation for plain packaging measures in the Philippines as the DOH works toward introducing plain packaging for cigarette products. Both the Singapore and Thailand packs (plain packaging mock-ups) were considered by most respondents from both surveys to be more effective compared to the Philippine packs (current pack mock-up). This is despite Singapore in particular being considered the most esthetically pleasing among the three packs, which is an undesirable trait for cigarette packs and GHWs for deterring use (10). Unappealing and unattractive designs have been found to deter use and encourage cessation (10). This shows the potential impact and acceptability of plain cigarette packaging and larger GHWs in the Philippines. By incorporating each mock-up’s best aspects from the survey’s responses, a more nuanced plain packaging prototype can be created to cater specifically to the preferences of the Filipino people.

The results also provide local evidence for the DOH and pro-health policymakers to pursue relevant policies, to inform not only non-smokers and the young of the harms of smoking, to help smokers to quit smoking (25), and to reduce the power of cigarette packaging as a marketing tool (26). More countries have also implemented plain packaging and larger GHWs in the past decade like Australia, France, the United Kingdom, Canada, and in the region, Thailand, and Singapore (27).

### 4.1. Study limitations

It should be noted that given the diverse profiles of our survey respondents, many of them have not been exposed to plain packaging on tobacco products before and thus, their unfamiliarity with them might have affected their perceptions of their attributes compared to the Philippine mock-ups. Considerable effort was made in the household survey to reach a sample representative of the population; however, this was not possible in the online survey. This limitation should be recognized in the interpretation of the results of the survey. With the online survey designed to recruit respondents through a social media platform, the limitations of convenience sampling in social media-administered surveys should be noted. However, it is also worth highlighting that despite the difference in the recruitment of respondents, the online and household survey results show that even across smoking profiles, there are more similarities than variability in the perceptions of respondents on the GHWs and plain packaging mock-ups.

Similarly, without a baseline of data to compare these results within the country, it is difficult to show the changes in GHW effectiveness over time. This study can serve as a baseline if and when plain packaging and larger GHWs are eventually implemented in the Philippines. The only possible comparable data available is from previous GATS in the Philippines about smokers’ thoughts of quitting smoking when exposed to health warnings on cigarette packs.

We recognize the limitations of using research literature for high-income and other middle-income countries to rationalize the potential impact of plain packaging in the Philippines. However, there is value in using the current evidence base to conduct research on this topic in a lower middle-income country like the Philippines. Because of the limited implementation of plain packaging in low-and middle-income countries, data on its impact is also still limited (28). Hence, while not exactly a fair comparison, using the available international evidence regardless of its origin is important in creating a baseline for research on graphic health warnings and plain packaging in the Philippines. Building the evidence base will support further policy research and evaluation needs for these measures. The evidence from the policy research will be useful in countering tobacco industry challenges to future reforms in graphic health warnings and plain packaging proposals in the Philippines (28).

Finally, our study cannot validate the relationship between GHWs and quitting motivations or motivation as a subjective measure without observed or reported behavior change. Since the survey is cross-sectional and can only capture perceptions at the time of the survey, we cannot claim or report any behavior change among our survey respondents compared to, for example, if this was a longitudinal study. Designing behavioral interventions that target behavior change vary in smoking cessation programs, including incorporating activities to improve motivation to quit, determining barriers to quitting, and counseling (29). In a review of smoking cessation programs with behavioral support, incorporating counseling and financial incentives were found to be of benefit to quitting smoking (30). To add, behavioral economics has been introduced and promoted in improving smoking cessation initiatives and tobacco control measures (30). Although not widely adopted in tobacco control, behavioral economics can also increase motivation toward a behavior change (e.g., quitting smoking). It can further improve the traditional interventions (e.g., smoking cessation) that are only moderately effective through a combination of interventions that utilize principles of psychology and economics (31).

## 5. Conclusion and recommendations

The results show that current GHW measures in the Philippines are ineffective, and policymakers should consider the implementation of larger graphic health warnings and the plain packaging of cigarettes to motivate smokers to quit and discourage Filipinos from smoking. Given the demographic nuances between the results of the online and household, it is recommended to consider the following minimum attributes for any plain packaging proposal in the Philippines: (a) the graphic health warning occupying 85% of the top sections of both the front and back of the pack, (b) brand name and variant limited to the lower bottom of the pack, (c) pack color in Pantone 448C, (d) rotating graphic health warnings with pilot testing before issuance, (e) visible text health warning on at least 60% of the sides of the pack, and; (f) visible quitline on the top sections of both sides of the pack. Further research should address the question of the viability of health warnings printed on cigarette sticks to combat GHW desensitization and consider the predominant by-stick cigarette purchasing behavior among the smoking population outside of the Philippines’ metropolitan region.

## Data availability statement

The datasets presented in this article are not readily available because of restrictions from data privacy laws and the approved research protocol for the study. Requests to access the datasets should be directed to ajsantiago@ateneo.edu.

## Ethics statement

The studies involving humans were approved by Ateneo de Manila University Research Ethics Office. The studies were conducted in accordance with the local legislation and institutional requirements. The participants provided their written informed consent to participate in this study.

## Author contributions

GA led the conceptualization of the paper. GA, EM, JA, and AS contributed to the drafting of the manuscript. GA and JA prepared the Results section. JA prepared the Introduction, Discussion, and Conclusion. EM prepared the Methodology part. All authors contributed to the article and approved the submitted version.
