# Supplementary material for: Graphic health warnings and plain packaging in the Philippines: results of online and household surveys
Source: Front Public Health. 2023 Sep 26;11:1207779. doi: 10.3389/fpubh.2023.1207779 (PMC10562603; doi:10.3389/fpubh.2023.1207779)
Supplement: Supplementary file 1 [file Table_1.DOCX]

Supplementary Material

Graphic health warnings and plain packaging in the Philippines: Results of online and household surveys

Gianna Gayle H. Amul, Eunice U. Mallari, John Rafael Y. Arda, Alen Josef A. Santiago

**Correspondence:** Gianna Gayle H. Amul*: [gamul@ateneo.edu](mailto:gamul@ateneo.edu)

# Supplementary Figures and Tables

| **Table 1. Socio-demographic profile of respondents per smoking status (n=93) for survey pilot testing** | | | | | |
| --- | --- | --- | --- | --- | --- |
|  | Smoking Status | | | | |
| Characteristics | I have never smoked | I smoke everyday | I smoke occasionally, at least once | I used to smoke but I don't smoke now | Total |
| **Sex** |  |  |  |  |  |
| Male | 27 | 1 | 2 | 8 | 38 |
| Female | 26 | 1 | 5 | 13 | 45 |
| Gender diverse | 7 | 0 | 0 | 2 | 9 |
| Not indicated | 1 | 0 | 0 | 0 | 1 |
| **Age Group** |  |  |  |  |  |
| 18-24 | 5 | 0 | 0 | 1 | 6 |
| 25-34 | 26 | 0 | 4 | 11 | 41 |
| 35-44 | 10 | 1 | 1 | 3 | 15 |
| 45-54 | 10 | 0 | 0 | 4 | 14 |
| 55-64 | 5 | 1 | 2 | 3 | 11 |
| 65-74 | 2 | 0 | 0 | 1 | 3 |
| Not indicated | 3 | 0 | 0 | 0 | 3 |
| **Region** |  |  |  |  |  |
| NCR | 39 | 1 | 7 | 14 | 61 |
| CAR | 1 | 0 | 0 | 0 | 1 |
| I | 1 | 0 | 0 | 0 | 1 |
| II | 0 | 0 | 0 | 0 | 0 |
| III | 2 | 1 | 0 | 0 | 3 |
| IV-A | 15 | 0 | 0 | 9 | 24 |
| IV-B | 0 | 0 | 0 | 0 | 0 |
| V | 1 | 0 | 0 | 0 | 1 |
| VII | 1 | 0 | 0 | 0 | 1 |
| VIII | 0 | 0 | 0 | 0 | 0 |
| IX | 0 | 0 | 0 | 0 | 0 |
| X | 0 | 0 | 0 | 0 | 0 |
| XI | 0 | 0 | 0 | 0 | 0 |
| XII | 0 | 0 | 0 | 0 | 0 |
| XIII | 0 | 0 | 0 | 0 | 0 |
| BARMM | 0 | 0 | 0 | 0 | 0 |
| Not indicated | 1 | 0 | 0 | 0 | 1 |
| **Educational Attainment** |  |  |  |  |  |
| No grade completed | 0 | 0 | 0 | 0 | 0 |
| Elementary undergraduate | 0 | 0 | 0 | 0 | 0 |
| Elementary graduate | 0 | 0 | 0 | 0 | 0 |
| Highschool undergraduate | 0 | 0 | 0 | 0 | 0 |
| Highschool graduate | 0 | 0 | 0 | 0 | 0 |
| Post-secondary/Vocational | 0 | 0 | 0 | 0 | 0 |
| College undergraduate | 4 | 0 | 0 | 1 | 5 |
| College graduate | 23 | 1 | 6 | 16 | 46 |
| Post-graduate | 33 | 1 | 1 | 6 | 41 |
| Not indicated | 1 | 0 | 0 | 0 | 1 |
| **Occupation** |  |  |  |  |  |
| Government | 9 | 0 | 2 | 3 | 14 |
| Non-government/Private | 41 | 2 | 5 | 16 | 64 |
| Self-employed | 6 | 0 | 0 | 2 | 8 |
| Student | 3 | 0 | 0 | 0 | 3 |
| Housekeeper | 0 | 0 | 0 | 0 | 0 |
| Retired | 1 | 0 | 0 | 0 | 1 |
| Unemployed, able to work | 0 | 0 | 0 | 2 | 2 |
| Unemployed, unable to work | 0 | 0 | 0 | 0 | 0 |
| Not indicated | 1 | 0 | 0 | 0 | 1 |
| **Montly Income** |  |  |  |  |  |
| a) No income | 2 | 0 | 0 | 1 | 3 |
| b) Below 3,499 | 1 | 0 | 0 | 0 | 1 |
| c) 3,500 to 4,999 | 1 | 0 | 0 | 0 | 1 |
| d) 5,000 to 8,499 | 2 | 0 | 0 | 1 | 3 |
| e) 8,500 to 19,999 | 4 | 0 | 1 | 3 | 8 |
| f) 20,000 to 20,999 | 2 | 0 | 0 | 0 | 2 |
| g) 21,000 to 29,999 | 7 | 0 | 2 | 3 | 12 |
| h) 30,000 to 39,999 | 4 | 0 | 0 | 6 | 10 |
| i) 40,000 - 49,999 | 11 | 0 | 1 | 1 | 13 |
| j) 50,000 or higher | 21 | 2 | 3 | 9 | 35 |
| Not indicated | 4 | 0 | 0 | 1 | 5 |

| **Table 2. Socio-demographic profile of online survey respondents per smoking status (n=1500)** | | | | | | | | | |
| --- | --- | --- | --- | --- | --- | --- | --- | --- | --- |
| Characteristics | Daily smokers | | Occasional smokers | | Former smokers |  | Never Smokers | | Total |
|  | n | (%) | n | (%) | n | (%) | n | (%) |  |
| **Sex** |  |  |  |  |  |  |  |  |  |
| Male | 170 | 53 | 72 | 30.1 | 141 | 43.8 | 194 | 31.4 | 577 |
| Female | 104 | 32.4 | 104 | 43.5 | 127 | 39.4 | 344 | 55.7 | 679 |
| Gender diverse | 47 | 14.6 | 63 | 26.4 | 54 | 16.8 | 80 | 12.9 | 244 |
| **Age Group** |  |  |  |  |  |  |  |  |  |
| 18-24 | 128 | 39.9 | 151 | 63.2 | 157 | 48.8 | 449 | 72.7 | 885 |
| 25-34 | 116 | 36.1 | 69 | 28.9 | 106 | 32.9 | 115 | 18.6 | 406 |
| 35-44 | 44 | 13.7 | 16 | 6.7 | 31 | 9.6 | 29 | 4.7 | 120 |
| 45-54 | 18 | 5.6 | 3 | 1.3 | 17 | 5.3 | 17 | 2.8 | 55 |
| 55-64 | 15 | 4.7 | 0 | 0 | 10 | 3.1 | 8 | 1.3 | 33 |
| 65-74 | 0 | 0 | 0 | 0 | 1 | 0.3 | 0 | 0 | 1 |
| **Region** |  |  |  |  |  |  |  |  |  |
| NCR | 139 | 43.3 | 107 | 44.8 | 146 | 45.3 | 246 | 39.8 | 638 |
| CAR | 7 | 2.2 | 6 | 2.5 | 5 | 1.6 | 10 | 1.6 | 28 |
| I | 10 | 3.1 | 11 | 4.6 | 6 | 1.9 | 17 | 2.8 | 44 |
| II | 5 | 1.6 | 2 | 0.8 | 6 | 1.9 | 12 | 1.9 | 25 |
| III | 33 | 10.3 | 22 | 9.2 | 38 | 11.8 | 71 | 11.5 | 164 |
| IV-A | 75 | 23.4 | 57 | 23.8 | 61 | 18.9 | 119 | 19.3 | 312 |
| IV-B | 1 | 0.3 | 0 | 0 | 1 | 0.3 | 7 | 1.1 | 9 |
| V | 8 | 2.5 | 8 | 3.3 | 11 | 3.4 | 18 | 2.9 | 45 |
| VI | 12 | 3.7 | 6 | 2.5 | 5 | 1.6 | 19 | 3.1 | 42 |
| VII | 11 | 3.4 | 7 | 2.9 | 18 | 5.6 | 37 | 6 | 73 |
| VIII | 2 | 0.6 | 0 | 0 | 6 | 1.9 | 7 | 1.1 | 15 |
| IX | 2 | 0.6 | 0 | 0 | 1 | 0.3 | 4 | 0.6 | 7 |
| X | 7 | 2.2 | 4 | 1.7 | 7 | 2.2 | 16 | 2.6 | 34 |
| XI | 5 | 1.6 | 4 | 1.7 | 5 | 1.6 | 17 | 2.8 | 31 |
| XII | 1 | 0.3 | 4 | 1.7 | 2 | 0.6 | 9 | 1.5 | 16 |
| XIII | 2 | 0.6 | 1 | 0.4 | 2 | 0.6 | 7 | 1.1 | 12 |
| BARMM | 1 | 0.3 | 0 | 0 | 2 | 0.6 | 2 | 0.3 | 5 |
| **Educational Attainment** |  |  |  |  |  |  |  |  |  |
| No grade completed | 0 | 0 | 1 | 0.4 | 0 | 0 | 1 | 0.2 | 2 |
| Elementary undergraduate | 0 | 0 | 0 | 0 | 0 | 0 | 1 | 0.2 | 1 |
| Elementary graduate | 0 | 0 | 1 | 0.4 | 1 | 0.3 | 1 | 0.2 | 3 |
| Highschool undergraduate | 8 | 2.5 | 5 | 2.1 | 9 | 2.8 | 55 | 8.9 | 77 |
| Highschool graduate | 24 | 7.5 | 26 | 10.9 | 28 | 8.7 | 108 | 17.5 | 186 |
| Post-secondary/Vocational | 9 | 2.8 | 4 | 1.7 | 9 | 2.8 | 8 | 1.3 | 30 |
| College undergraduate | 106 | 33 | 86 | 36 | 93 | 28.9 | 194 | 31.4 | 479 |
| College graduate | 141 | 43.9 | 101 | 42.3 | 155 | 48.1 | 204 | 33 | 601 |
| Post-graduate | 33 | 10.3 | 15 | 6.3 | 27 | 8.4 | 46 | 7.4 | 121 |
| **Occupation** |  |  |  |  |  |  |  |  |  |
| Government | 36 | 11.2 | 20 | 8.4 | 24 | 7.5 | 59 | 9.5 | 139 |
| Non-government/Private | 138 | 43 | 80 | 33.5 | 116 | 36 | 118 | 19.1 | 452 |
| Self-employed | 51 | 15.9 | 16 | 6.7 | 43 | 13.4 | 41 | 6.6 | 151 |
| Student | 63 | 19.6 | 102 | 42.7 | 108 | 33.5 | 361 | 58.4 | 634 |
| Housekeeper | 3 | 0.9 | 0 | 0 | 3 | 0.9 | 1 | 0.2 | 7 |
| Retired | 7 | 2.2 | 0 | 0 | 2 | 0.6 | 1 | 0.2 | 10 |
| Unemployed, able to work | 23 | 7.2 | 20 | 8.4 | 24 | 7.5 | 31 | 5 | 98 |
| Unemployed, unable to work | 0 | 0 | 1 | 0.4 | 2 | 0.6 | 6 | 1 | 9 |
| **Monthly income (PhP)** |  |  |  |  |  |  |  |  |  |
| <10,957 | 40 | 12.5 | 36 | 15.1 | 39 | 12.1 | 68 | 11 | 183 |
| 10,957-21,914 | 64 | 19.9 | 55 | 23 | 43 | 13.4 | 87 | 14.1 | 249 |
| 21,914-43,828 | 103 | 32.1 | 41 | 17.2 | 84 | 26.1 | 95 | 15.4 | 323 |
| 43,828-76,669 | 36 | 11.2 | 20 | 8.4 | 31 | 9.6 | 23 | 3.7 | 110 |
| 76,669-131,484 | 8 | 2.5 | 9 | 3.8 | 19 | 5.9 | 21 | 3.4 | 57 |
| 131,483-219,140 | 4 | 1.2 | 2 | 0.8 | 2 | 0.6 | 5 | 0.8 | 13 |
| >219,140 | 4 | 1.2 | 2 | 0.8 | 6 | 1.9 | 8 | 1.3 | 20 |
| No income | 62 | 19.3 | 74 | 31 | 98 | 30.4 | 311 | 50.3 | 545 |
| Total | 321 | 100 | 239 | 100 | 322 | 100 | 618 | 100 | 1500 |

| **Table 3. Socio-demographic profile of household survey respondents per smoking status (n=1201**) | | | | | | | | | |
| --- | --- | --- | --- | --- | --- | --- | --- | --- | --- |
| Characteristics | Daily smokers | | Occasional smokers | | Former Smokers | | Never Smokers | | Total |
|  | n | (%) | n | (%) | n | (%) | n | (%) |  |
| **Sex** |  |  |  |  |  |  |  |  |  |
| Male | 315 | 81 | 61 | 69.3 | 142 | 70.6 | 107 | 20.2 | 625 |
| Female | 68 | 17.5 | 24 | 27.3 | 54 | 26.9 | 416 | 78.3 | 562 |
| Gender diverse | 7 | 1.8 | 3 | 3.4 | 0 | 0 | 4 | 0.8 | 14 |
| **Age Group** |  |  |  |  |  |  |  |  |  |
| 18-24 | 47 | 12.1 | 12 | 13.6 | 6 | 3 | 63 | 11.9 | 128 |
| 25-34 | 94 | 24.2 | 16 | 18.2 | 26 | 12.9 | 124 | 23.4 | 260 |
| 35-44 | 109 | 28 | 21 | 23.9 | 38 | 18.9 | 112 | 21.1 | 280 |
| 45-54 | 77 | 19.8 | 22 | 25 | 50 | 24.9 | 134 | 25.2 | 283 |
| 55-64 | 58 | 14.9 | 14 | 15.9 | 62 | 30.8 | 78 | 14.7 | 212 |
| 65-74 | 5 | 1.3 | 3 | 3.4 | 14 | 7 | 16 | 3 | 38 |
| **Region** |  |  |  |  |  |  |  |  |  |
| NCR | 96 | 24.7 | 18 | 20.5 | 44 | 21.9 | 142 | 26.7 | 300 |
| I | 19 | 4.9 | 1 | 1.1 | 15 | 7.5 | 24 | 4.5 | 59 |
| II | 0 | 0 | 0 | 0 | 1 | 0.5 | 0 | 0 | 1 |
| III | 36 | 9.3 | 6 | 6.8 | 8 | 4 | 11 | 2.1 | 61 |
| IV-A | 32 | 8.2 | 9 | 10.2 | 18 | 9 | 61 | 11.5 | 120 |
| V | 45 | 11.6 | 6 | 6.8 | 3 | 1.5 | 9 | 1.7 | 63 |
| VI | 33 | 8.5 | 21 | 23.9 | 30 | 14.9 | 34 | 6.4 | 118 |
| VII | 18 | 4.6 | 4 | 4.5 | 10 | 5 | 29 | 5.5 | 61 |
| VIII | 21 | 5.4 | 4 | 4.5 | 13 | 6.5 | 83 | 15.6 | 121 |
| IX | 17 | 4.4 | 3 | 3.4 | 9 | 4.5 | 30 | 5.6 | 59 |
| X | 25 | 6.4 | 4 | 4.5 | 12 | 6 | 19 | 3.6 | 60 |
| XI | 13 | 3.3 | 4 | 4.5 | 16 | 8 | 27 | 5.1 | 60 |
| XIII | 35 | 9 | 8 | 9.1 | 17 | 8.5 | 58 | 10.9 | 118 |
| **Educational Attainment** |  |  |  |  |  |  |  |  |  |
| No grade completed | 5 | 1.3 | 1 | 1.1 | 0 | 0 | 3 | 0.6 | 9 |
| Preschool | 2 | 0.5 | 0 | 0 | 0 | 0 | 0 | 0 | 2 |
| Elementary undergraduate | 46 | 11.8 | 10 | 11.4 | 28 | 13.9 | 44 | 8.3 | 128 |
| Elementary graduate | 58 | 14.9 | 9 | 10.2 | 33 | 16.4 | 45 | 8.5 | 145 |
| Highschool undergraduate | 80 | 20.6 | 22 | 25 | 34 | 16.9 | 80 | 15.1 | 216 |
| Highschool graduate | 105 | 27 | 28 | 31.8 | 52 | 25.9 | 198 | 37.3 | 383 |
| Post-secondary/Vocational | 17 | 4.4 | 5 | 5.7 | 6 | 3 | 19 | 3.6 | 47 |
| College undergraduate | 37 | 9.5 | 7 | 8 | 22 | 10.9 | 73 | 13.7 | 139 |
| College graduate | 40 | 10.3 | 6 | 6.8 | 20 | 10 | 64 | 12.1 | 130 |
| Post-graduate | 0 | 0 | 0 | 0 | 1 | 0.5 | 1 | 0.2 | 2 |
| **Occupation** |  |  |  |  |  |  |  |  |  |
| Government | 55 | 14.1 | 13 | 14.8 | 31 | 15.4 | 79 | 14.9 | 178 |
| Non-government/Private | 86 | 22.1 | 11 | 12.5 | 29 | 14.4 | 57 | 10.7 | 183 |
| Self-employed | 120 | 30.8 | 35 | 39.8 | 72 | 35.8 | 133 | 25 | 360 |
| Student | 14 | 3.6 | 5 | 5.7 | 0 | 0 | 31 | 5.8 | 50 |
| Housekeeper | 34 | 8.7 | 5 | 5.7 | 22 | 10.9 | 164 | 30.9 | 225 |
| Retired | 0 | 0 | 0 | 0 | 4 | 2 | 3 | 0.6 | 7 |
| Unemployed, able to work | 68 | 17.5 | 18 | 20.5 | 28 | 13.9 | 52 | 9.8 | 166 |
| Unemployed, unable to work | 13 | 3.3 | 1 | 1.1 | 10 | 5 | 8 | 1.5 | 32 |
| **Monthly income (PhP)** |  |  |  |  |  |  |  |  |  |
| <10,957 | 208 | 53.5 | 50 | 56.8 | 114 | 56.7 | 264 | 49.7 | 636 |
| 10,957-21,914 | 45 | 11.6 | 9 | 10.2 | 17 | 8.5 | 38 | 7.2 | 109 |
| 21,914-43,828 | 6 | 1.5 | 0 | 0 | 3 | 1.5 | 8 | 1.5 | 17 |
| 43,828-76,669 | 4 | 1 | 0 | 0 | 0 | 0 | 1 | 0.2 | 5 |
| 76,669-131,484 | 0 | 0 | 0 | 0 | 1 | 0.5 | 1 | 0.2 | 2 |
| 131,483-219,140 | 0 | 0 | 0 | 0 | 0 | 0 | 0 | 0 | 0 |
| >219,140 | 1 | 0.3 | 0 | 0 | 1 | 0.5 | 0 | 0 | 2 |
| No income | 126 | 32.4 | 26 | 29.5 | 63 | 31.3 | 215 | 40.5 | 430 |
| Total | 389 | 100 | 88 | 100 | 201 | 100 | 531 | 100 | 1201 |

| **Table 4. Perceptions towards the current and mock-up packaging according to the smoking status of online survey respondents** | | | | | | | | | | | | |
| --- | --- | --- | --- | --- | --- | --- | --- | --- | --- | --- | --- | --- |
|  |  |  |  |  |  |  |  |  |  |  |  |  |
| **Attributes** | Strongly Agree/Agree (%) | | | | | | | | | | | |
|  | Daily Smokers | | | Occasional Smokers | | | Former Smokers | | | Never Smokers | | |
| *Pack Mock-up* | PH | SG | TH | PH | SG | TH | PH | SG | TH | PH | SG | TH |
| ***Pack Characteristics*** |  |  |  |  |  |  |  |  |  |  |  |  |
| Attractiveness | 22.9 | 43.9 | 31 | 23.2 | 45.4 | 34.2 | 33 | 43.2 | 30.9 | 35.7 | 50.5 | 38.2 |
| Quality | 62.8 | 47 | 34.3 | 55.7 | 58.7 | 38.7 | 53.9 | 51.8 | 31.9 | 37.3 | 48.7 | 32.3 |
| Taste | 58.1 | 47.2 | 42.2 | 61.4 | 58 | 56.5 | 58.8 | 55.9 | 45.7 | 45.4 | 53.3 | 47.3 |
| Cost | 62.7 | 58.3 | 39.1 | 51.3 | 65.9 | 44.8 | 59.7 | 61.9 | 41.3 | 56.9 | 62.7 | 40.5 |
| Social appeal | 29.4 | 32.8 | 24.3 | 23.6 | 40.9 | 21.7 | 26.9 | 27.1 | 15.6 | 14.3 | 22 | 14.2 |
| Appeal to youth | 27.4 | 30.8 | 19 | 32.3 | 41.5 | 23.1 | 35.1 | 32.9 | 24.1 | 37.6 | 32.4 | 23.5 |
|  |  |  |  |  |  |  |  |  |  |  |  |  |
| ***Graphic health warnings*** |  |  |  |  |  |  |  |  |  |  |  |  |
| Noticeability of health warnings | 78.3 | 82.4 | 78.4 | 82.3 | 86.1 | 79.7 | 77.6 | 83.9 | 78.2 | 73.6 | 82.8 | 79 |
| Appeal to non-smokers | 6.7 | 12.7 | 13.2 | 7.2 | 13.1 | 10.8 | 6.4 | 12.7 | 9.5 | 7 | 9.6 | 7.7 |
| Attempt to quit | 29.3 | 47.2 | 45.2 | 41.1 | 55.5 | 55.4 | 45.5 | 63.3 | 62.7 | 55.6 | 68.3 | 72.4 |
| Ease of quitting | 6.8 | 10.2 | 10.5 | 8.6 | 9.8 | 8.2 | 8.1 | 9.6 | 10.7 | 9.8 | 13.2 | 13.6 |

| **Table 5. Perceptions towards the current and mock-up packaging according to the smoking status of household survey respondents** | | | | | | | | | | | | |
| --- | --- | --- | --- | --- | --- | --- | --- | --- | --- | --- | --- | --- |
|  |  |  |  |  |  |  |  |  |  |  |  |  |
| Attributes | Strongly Agree/Agree % | | | | | | | | | | | |
|  | Daily Smokers | | | Occasional Smokers | | | Previous Smokers | | | Never Smokers | | |
| *Pack Mock-up* | PH | SG | TH | PH | SG | TH | PH | SG | TH | PH | SG | TH |
| ***Pack Characteristics*** |  |  |  |  |  |  |  |  |  |  |  |  |
| Attractiveness | 26.7 | 29.3 | 29.5 | 30.7 | 30.7 | 25 | 22.5 | 20.4 | 23 | 15.6 | 16.4 | 19.4 |
| Quality | 45 | 42.5 | 42.6 | 36.4 | 44.3 | 44.3 | 31.6 | 32.1 | 34.7 | 28 | 34.6 | 36.9 |
| Taste | 64.7 | 61 | 64.3 | 56.8 | 70.1 | 62.5 | 64.6 | 71.4 | 73.5 | 53.1 | 58.8 | 58.7 |
| Cost | 79.4 | 78.7 | 75.5 | 86.1 | 84.1 | 76.7 | 82.1 | 85.2 | 84.2 | 75.5 | 78.5 | 75.2 |
| Social appeal | 32.6 | 23.3 | 20.3 | 15.9 | 12.5 | 12.5 | 7.6 | 6.2 | 6.9 | 5 | 4.5 | 4.5 |
| Appeal to youth | 23.2 | 23.4 | 18.6 | 25 | 24.1 | 20.5 | 27.6 | 19.9 | 18.4 | 22.9 | 20.2 | 18.1 |
|  |  |  |  |  |  |  |  |  |  |  |  |  |
| ***Graphic health warnings*** |  |  |  |  |  |  |  |  |  |  |  |  |
| Noticeability of health warnings | 76.8 | 80.3 | 80.4 | 77 | 81.8 | 81.8 | 89.8 | 89.2 | 85.1 | 86.7 | 88.2 | 86 |
| Appeal to non-smokers | 30.2 | 19.8 | 21.7 | 18.8 | 11.4 | 9.9 | 9.7 | 9 | 5.7 | 3.3 | 4.4 | 4.8 |
| Attempt to quit | 58 | 64.8 | 66.8 | 69.1 | 70.6 | 76.7 | 71.1 | 77.3 | 82.5 | 55.6 | 46.9 | 45.8 |
| Ease of quitting | 34.5 | 33.1 | 34.2 | 29.4 | 35.6 | 37.5 | 29.6 | 31 | 33.3 | 37 | 35.5 | 33.3 |

**
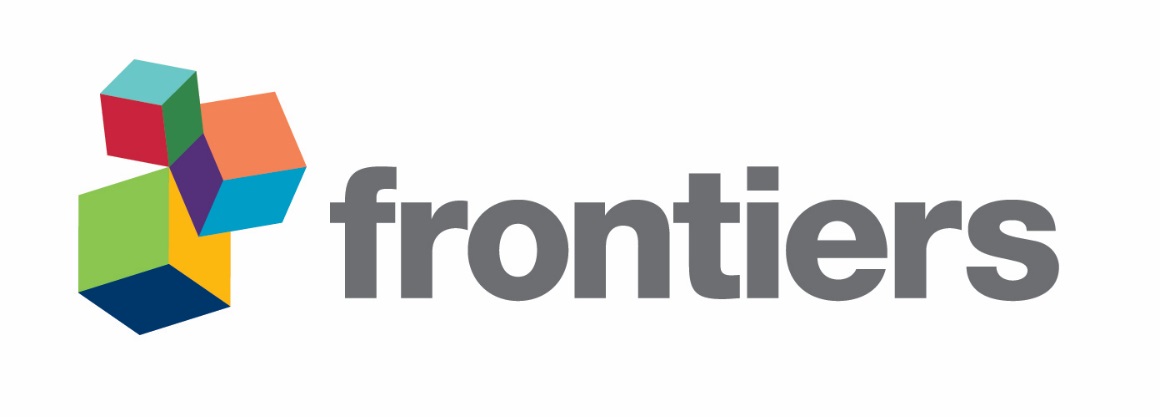
**
