# Supplementary material for: Graphic health warnings and plain packaging in the Philippines: results of online and household surveys
Source: Front Public Health. 2023 Sep 26;11:1207779. doi: 10.3389/fpubh.2023.1207779 (PMC10562603; doi:10.3389/fpubh.2023.1207779)
Supplement: Supplementary file 2 [file Data_Sheet_1.PDF]

What do Filipinos think of cigarette packaging?

You can help us find out! We are researchers from Ateneo de Manila University and our survey is about how graphic health warnings on cigarette packs have affected smoking behavior among Filipinos and explore the potential of plain packaging of cigarettes in the Philippines. The results will support the Department of Health in shaping policies for tobacco control.

To take part, here’s what you need to know:
- It takes about 5 to 10 minutes
- It’s completely voluntary
- You don't have to answer any question you don’t want to and you can stop at any time
- You and your survey responses will be anonymous to the researchers
- The survey responses will be stored in secure facilities, accessible to research team members only.

For more information: You can email policycenter.asog@ateneo.edu with any questions about the research.

Thinking of quitting? For questions about your own smoking habit, you could talk to your doctor or reach out to the Department of Health’s quitline: 165-364 or SMS 09212039534 or 09776277539.

This project has been reviewed by the Ateneo de Manila University’s Research Ethics Committee. Ms Ariza Francisco, Ms Gianna Gayle Amul and Ms Eunice Mallari are responsible for the ethical conduct of this research.

If you have any concerns about the conduct of this research that you want to raise with someone other than the researchers, please contact Ateneo IRB at (+63) 945 213 6758.

By proceeding to the survey, you voluntarily agree to participate and you confirm that you're 18 years old and above, and that you are not employed or have been employed by the tobacco industry.

You may read and download the full consent form via this link: http://bit.ly/39uSt5Z

* Required

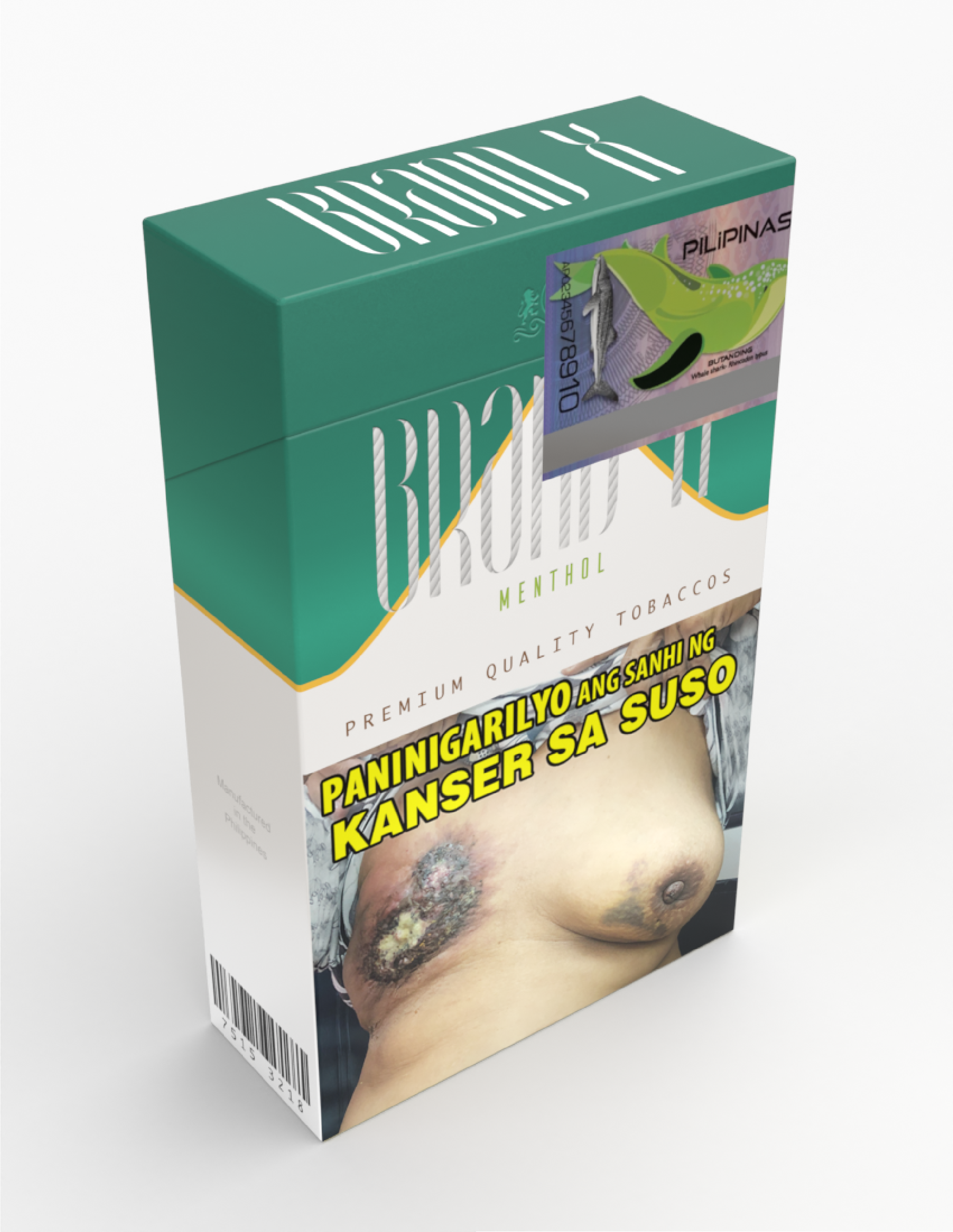

Section A: Demographics

We would first like to know your background.

1. Gender *

   Mark only one oval.

   - Female
   - Male
   - Gender diverse (Lesbian, Gay, Bisexual, Transgender, Queer (or those questioning their gender identity or sexual orientation), intersex, and asexual, or their allies)

2. Age: How old are you? *

   ____

3. Your location (according to region) *

Mark only one oval.

- a) National Capital Region
- b) Cordillera Administrative Region
- c) Region I (Ilocos Region)
- d) Region II (Cagayan Valley)
- e) Region III (Central Luzon)
- f) Region IV-A (CALABARZON)
- g) Region IV-B (MIMAROPA)
- h) Region V (Bicol Region)
- i) Region VI (Western Visayas)
- j) Region VII (Central Visayas)
- k) Region VIII (Eastern Visayas)
- l) Region IX (Zamboanga Peninsula)
- m) Region X (Northern Mindanao)
- n) Region XI (Davao Region)
- o) Region XII (Soccsksargen)
- p) Region XIII (CARAGA)
- q) BARMM

4. What is the highest level of education you have completed? (Select only one) *

Mark only one oval.

- a) No grade completed
- b) Preschool
- c) Elementary undergraduate
- d) Elementary graduate
- e) High school undergraduate
- f) High school graduate
- g) Post-secondary/non-tertiary/technical-vocational education graduate
- h) College undergraduate
- i) College graduate
- j) Post-graduate degree completed

5. Occupation: Which of the following best describes your main work status over the past 12 months? *

Mark only one oval.

- a) Government employee
- b) Non-government employee
- c) Self-employed
- d) Student
- e) Housekeeper
- f) Retired
- g) Unemployed, able to work
- h) Unemployed, unable to work

6. Monthly income: Can you let me know which category your monthly income falls under? *

Mark only one oval.

- No income
- Lower than PhP 10,957
- PhP 10,957 to PhP 21,914
- PhP 21,914 to PhP 43,828
- PhP 43,828 to PhP 76,669
- PhP 76,669 to PhP 131,484
- PhP 131,483 to PhP 219,140
- More than PhP 219,140

Section B: Smoking and Quitting Smoking

You will now be asked about smoking and quitting smoking.

7. Which of the following statements best describes you? *

Mark only one oval.

- I smoke everyday *Skip to question 9*
- I smoke occassionally, at least once a month, but not every day, depending on the event or the people I am with. *Skip to question 9*
- I used to smoke but I don't smoke now. *Skip to question 15*
- I have never smoked *Skip to question 8*

I have never smoked

8. Which of the following statement best describes you? *

Mark only one oval.

- I don't smoke and have never smoked a cigarette *Skip to question 18*
- I don’t smoke and have never smoked a cigarette, I only use an ecigarette/vape *Skip to question 16*

9. When did you start smoking? (Year) *

____

10. Do you buy cigarettes... *

Mark only one oval.

- by the stick?
- by the pack?

11. Do you also use e-cigarettes? (dual use) *

Mark only one oval.

- No *Skip to question 14*
- Yes *Skip to question 12*

e-cigarette use

12. If yes, how often do you use e-cigarettes? *

Mark only one oval.

- 1-2 times a day
- 3-4 times a day
- 5 or more times a day

13. If yes, what do you use on your ENDS device (electronic nicotine delivery system)? *

Mark only one oval.

- e-liquids
- freebase nicotine
- Other: ____________________

Quitting Smoking

14. Which of the following best describes your thinking about quitting smoking? *

Mark only one oval.

- (a) I have thought about quitting but not seriously and haven't cut down or tried to
- (b) I have thought seriously about wanting to quit in the next six months but I haven't done anything yet
- (c) I intend to quit in the next six months and taking the steps to do so, I am currently in the process of quitting/cutting down
- (d) I have tried quitting but keep starting again
- (e) I have not thought of quitting at all

Skip to question 18

I used to smoke...

15. Which of the following statement best describes you? *

Mark only one oval.

- I have recently quit (less than 12 months) *Skip to question 18*
- I recently quit smoking and are currently using an e-cigarette/vape *Skip to question 16*
- I have quit smoking for good (quit in the last 10 years) *Skip to question 18*

e-cigarette use

16. If yes, how often do you use e-cigarettes? *

Mark only one oval.

- 1-2 times a day
- 3-4 times a day
- 5 or more times a day

17. If yes, what do you use on your ENDS device (electronic nicotine delivery system)? *

Mark only one oval.

- e-liquids
- freebase nicotine
- Other: ______________________

Section C: Graphic health warnings and plain packaging

Next, we are going to ask you about your exposure to graphic health warnings on cigarette packages.

18. In the last 30 days, did you notice any health warnings on cigarette packages? *

Mark only one oval.

- Yes
- No
- I did not see any cigarette packages

19. In the last 30 days, have warning labels on cigarette packages led you to think about quitting? *

Mark only one oval.

- Yes
- No
- I did not see any cigarette packages

Pack A (Philippines)

For the following sections, please look at the specified pack and answer the questions
about the pack and the graphic health warning labels on the specified cigarette pack. Each
pack has a different design and graphic health warning that we would like you to look at.

The cigarette pack design and image used here is based on the Philippines current packaging regulations.

20. Look at Pack A, to what extent would you agree that: *

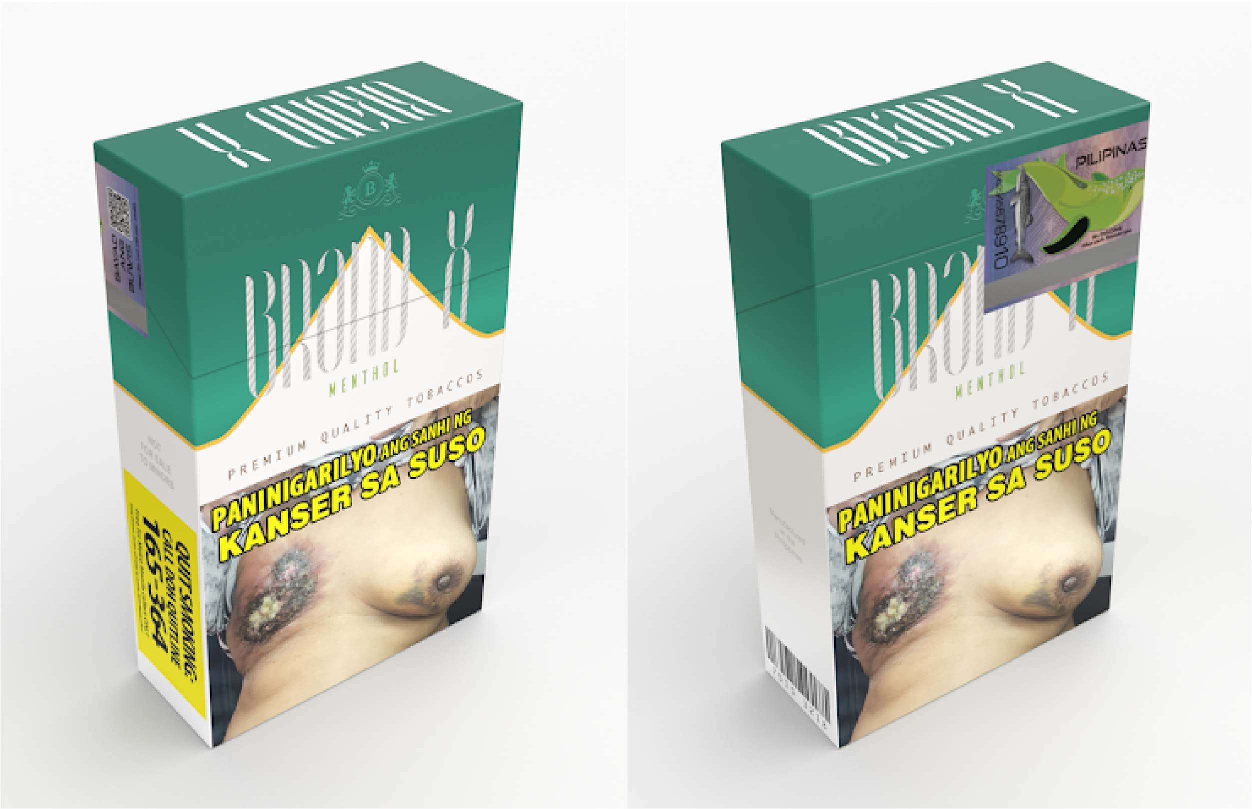

Mark only one oval per row.

	Strongly disagree	Disagree	Neither agree or disagree	Agree	Strongly Agree	Not applicable
**this pack has an attractive design/ pleasing to the eyes/ catches the eye?**						
**this pack contains cigarettes of a high quality?**						
**this pack contains cigarettes with a strong taste?**						
**this pack is expensive?**						

**this is a pack that you would like to be seen smoking?**	⬭	⬭	⬭	⬭	⬭	⬭
**this is a pack likely to attract youths below 18 to try?**	⬭	⬭	⬭	⬭	⬭	⬭
**the graphic health warning on the pack is noticeable/stands out visually on the pack?**	⬭	⬭	⬭	⬭	⬭	⬭
**the graphic health warning on the pack is likely to encourage you to TRY smoking?**	⬭	⬭	⬭	⬭	⬭	⬭
**the graphic health warning on the pack encourages you to try to QUIT smoking?**	⬭	⬭	⬭	⬭	⬭	⬭
**the graphic health warning on the pack makes it hard for you to QUIT smoking?**	⬭	⬭	⬭	⬭	⬭	⬭

21. Look at Pack A again, when you saw the graphic health warning... *

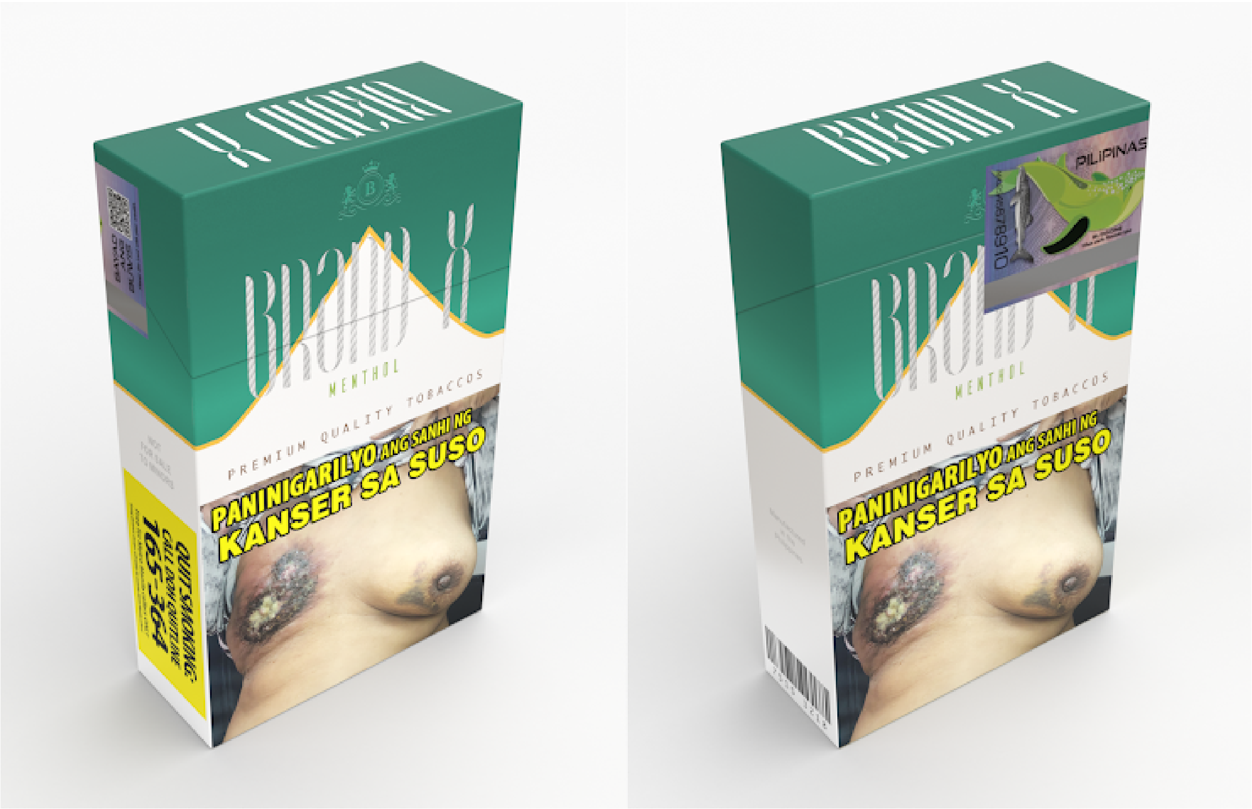

Mark only one oval per row.

	Not at all	Slightly	Very	Extremely	Not Applicable
**did you think that you should NOT smoke?**					
**did you think that smoking is harmful to health?**					

22. In addition to the above, whether you're a smoker or not, we'd like to know if you have additional comments/feedback about this cigarette pack.

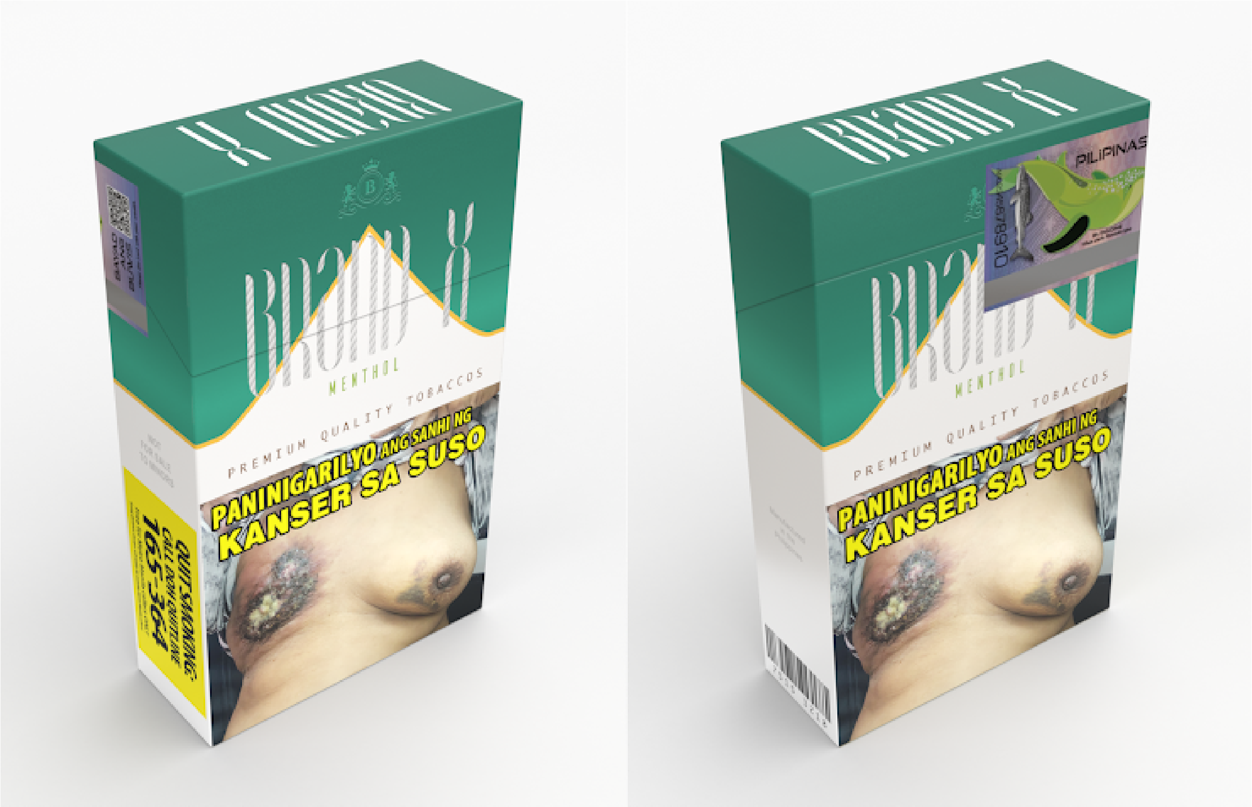

Pack B (Singapore)

Reminder: For the following sections, please look at the specified pack and answer the
questions about the pack and the graphic health warning labels on the specified cigarette
pack. Each pack has a different design and graphic health warning that we would like you
to look at.

The cigarette pack design and image used here is based on Singapore's standardized packaging regulations.

23. Look at Pack B, to what extent would you agree that: *

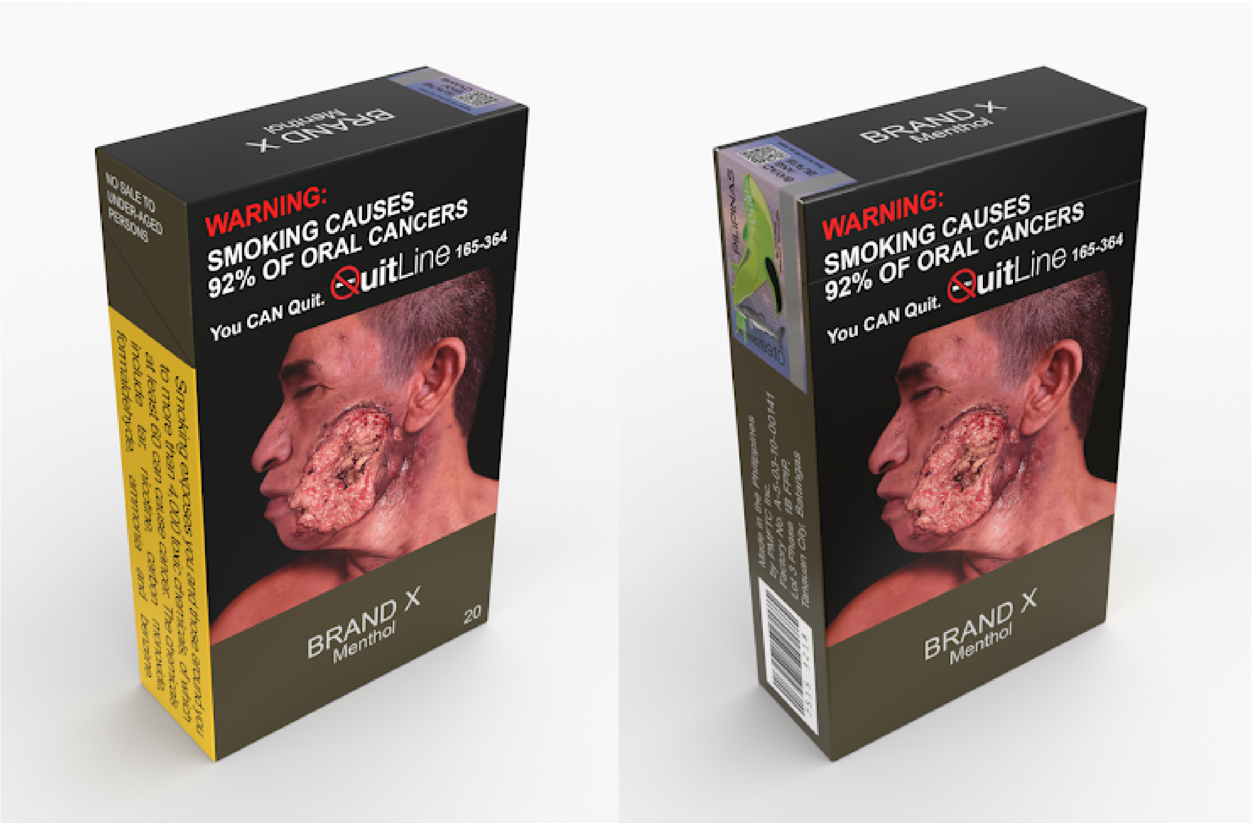

Mark only one oval per row.

	Strongly disagree	Disagree	Neither agree or disagree	Agree	Strongly Agree	Not applicable
**this pack has an attractive design/pleasing to the eyes/ catches the eye?**						
**this pack contains cigarettes of a high quality?**						
**this pack contains cigarettes with a strong taste?**						
**this pack is expensive?**						

**this is a pack that you would like to be seen smoking?**						
**this pack is likely to attract youths below 18 to try?**						
**the graphic health warning on the pack is noticeable/ stands out visually on the pack?**						
**the graphic health warning on the pack is likely to encourage you to TRY smoking?**						
**the graphic health warning on the pack encourages you to try to QUIT smoking?**						
**the graphic health warning on the pack makes it hard for you to QUIT smoking?**						

24. Look at Pack B again, when you saw the graphic health warning... *

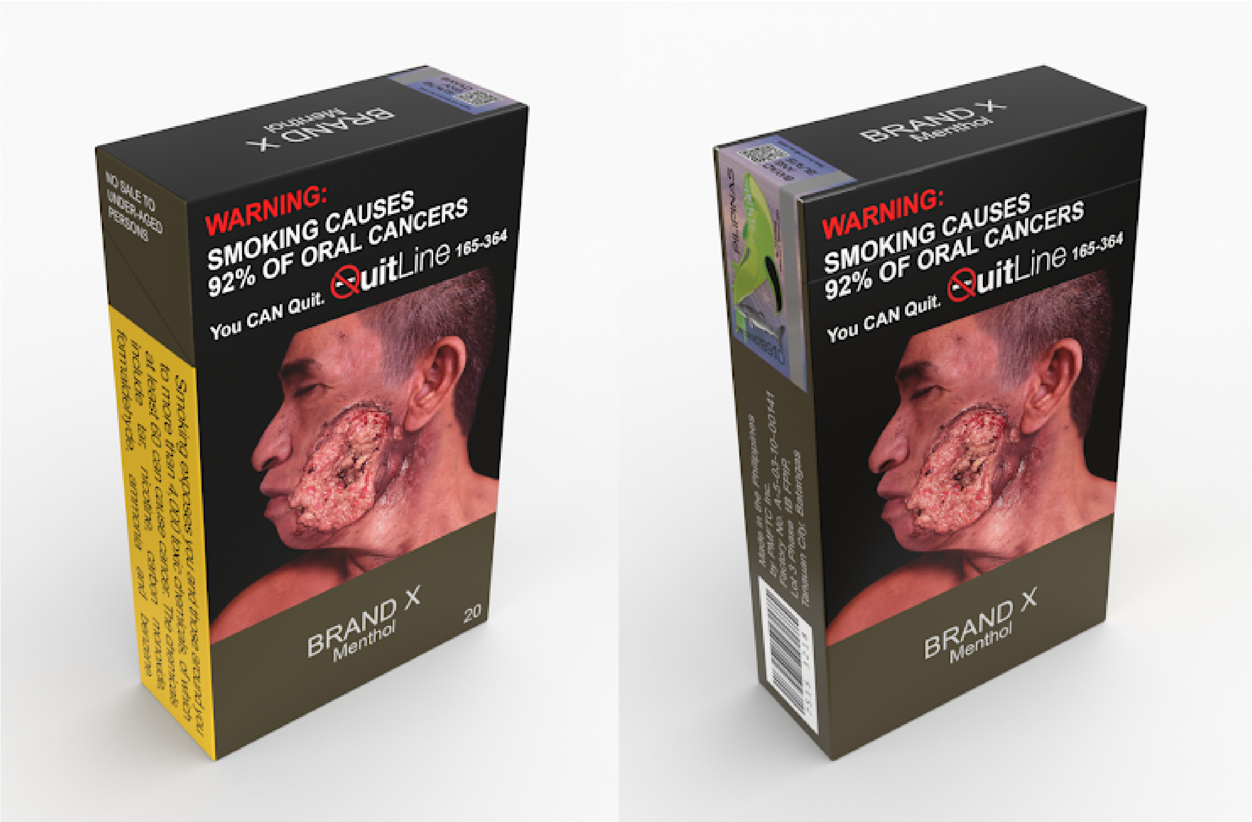

Mark only one oval per row.

	Not at all	Slightly	Very	Extremely	Not applicable
**did you think that you should not smoke?**	⬭	⬭	⬭	⬭	⬭
**did you think that smoking is harmful to health?**	⬭	⬭	⬭	⬭	⬭

25. In addition to the above, whether you're a smoker or not, we'd like to know if you have additional comments/feedback about this cigarette pack.

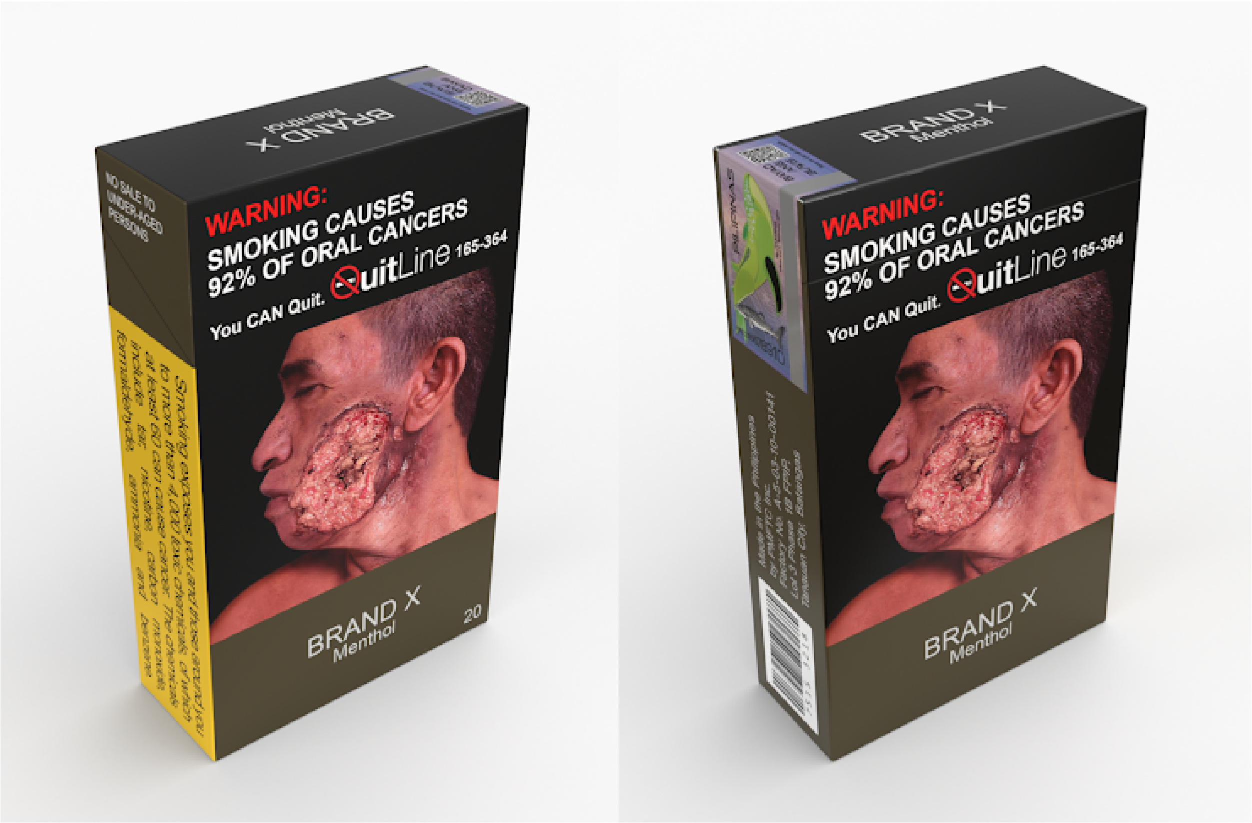

Pack C (Thailand)

Reminder: For the following sections, please look at the specified pack and answer the
questions about the pack and the graphic health warning labels on the specified cigarette
pack. Each pack has a different design and graphic health warning that we would like you
to look at.

The cigarette pack design and image used here is based on Thailand's plain packaging regulations.

26. Look at Pack C, to what extent would you agree that:

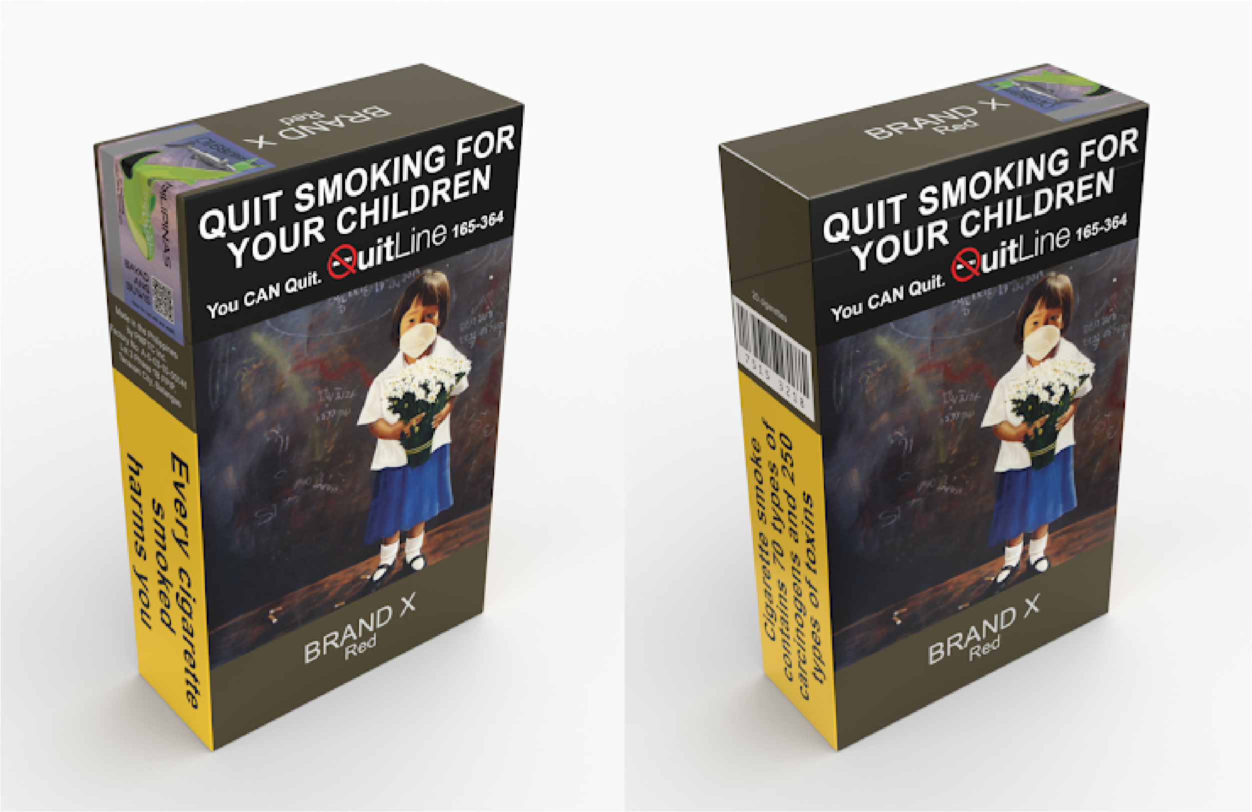

Mark only one oval per row.

	Strongly disagree	Disagree	Neither agree or disagree	Agree	Strongly Agree	Not applicable
**this pack has an attractive design/ pleasing to the eyes/ catches the eye?**						
**this pack contains cigarettes of a high quality?**						
**this pack contains cigarettes with a**						

strong taste?						
this pack is expensive?	◯	◯	◯	◯	◯	◯
this is a pack that you would like to be seen smoking?	◯	◯	◯	◯	◯	◯
this pack is likely to attract youths below 18 to try?	◯	◯	◯	◯	◯	◯
the graphic health warning on the pack is noticeable/ stands out visually on the pack?	◯	◯	◯	◯	◯	◯
the graphic health warning on the pack is likely to encourage you to TRY smoking?	◯	◯	◯	◯	◯	◯
the graphic health warning on the pack encourages you to try to QUIT smoking?	◯	◯	◯	◯	◯	◯

**the graphic health warning on the pack makes it hard for you to QUIT smoking?**	◯	◯	◯	◯	◯	◯

27. Look at Pack C again, when you saw the graphic health warning... *

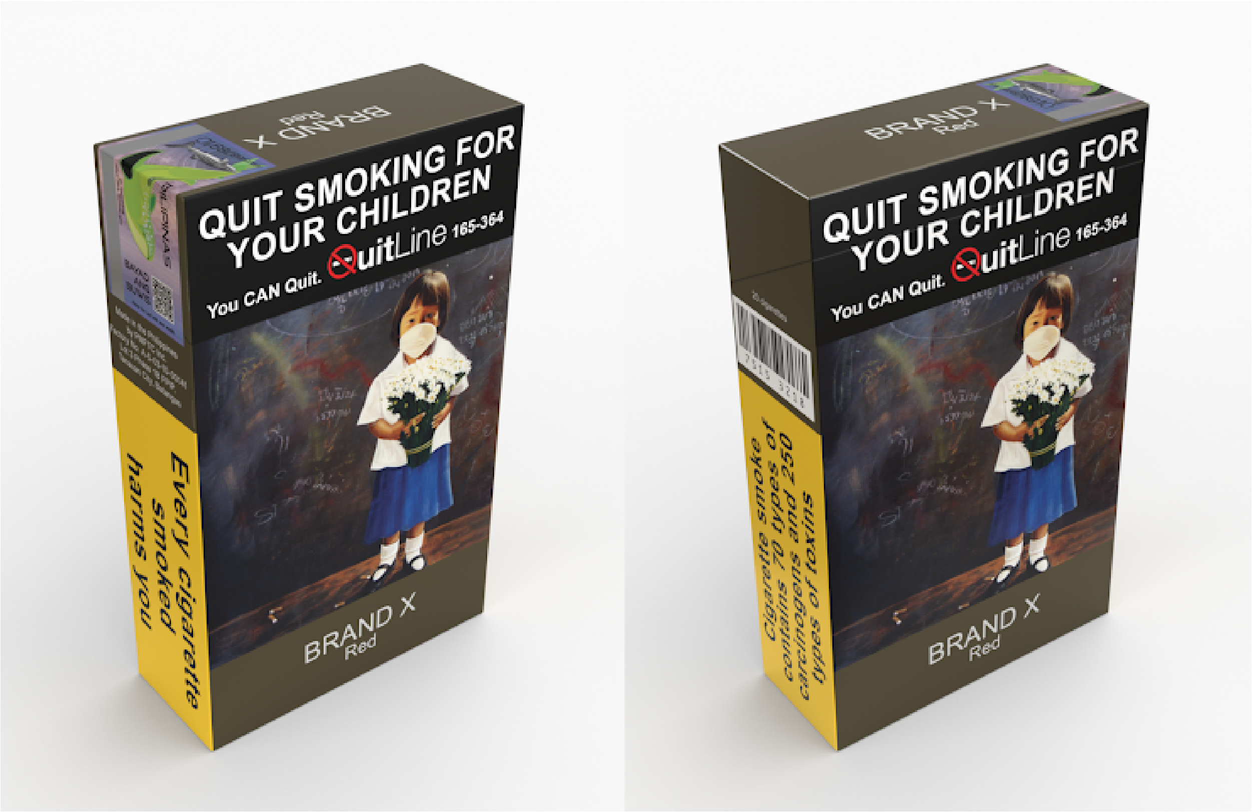

Mark only one oval per row.

	Not at all	Slightly	Very	Extremely	Not applicable
**did you think that you should not smoke?**	⬭	⬭	⬭	⬭	⬭
**did you think that smoking is harmful to health?**	⬭	⬭	⬭	⬭	⬭

28. In addition to the above, whether you're a smoker or not, we'd like to know if you have additional comments/feedback about this cigarette pack.

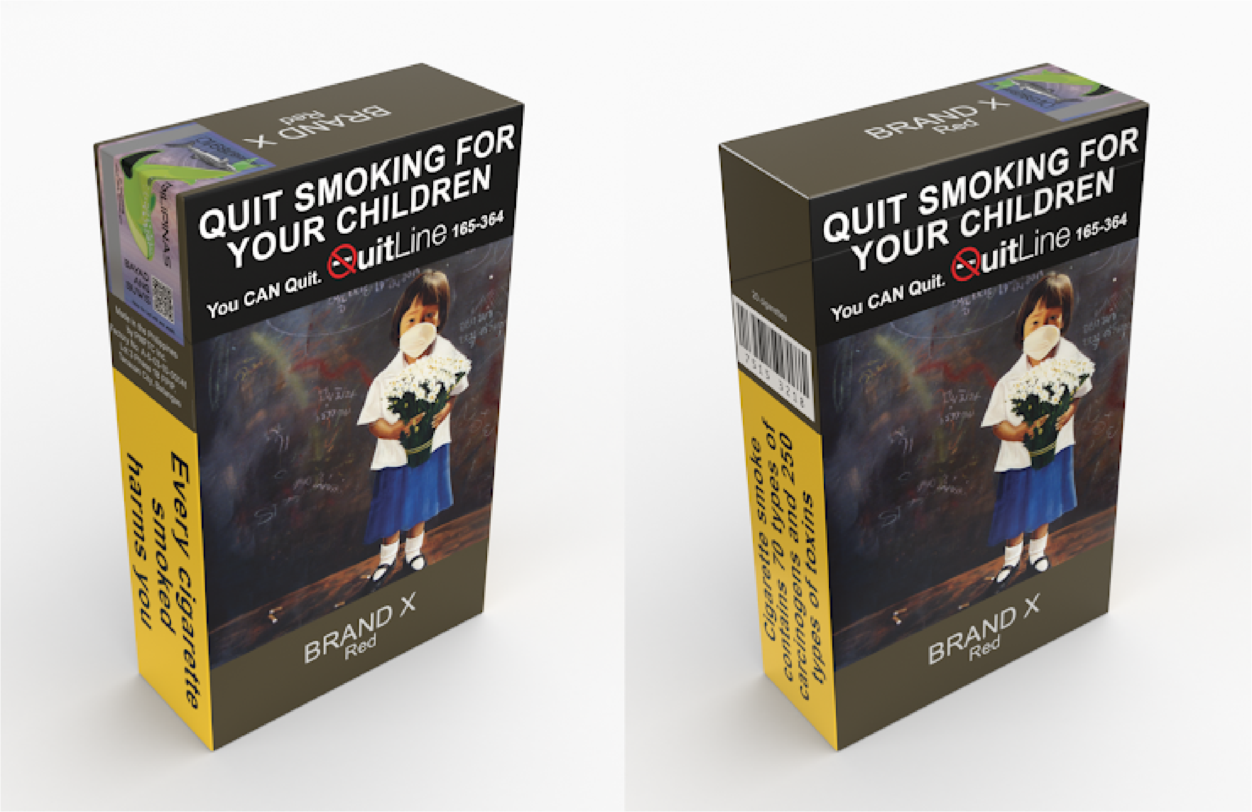

29. In your opinion, which of the three do you think is the most effective in convincing you to NOT SMOKE? *

Mark only one oval.

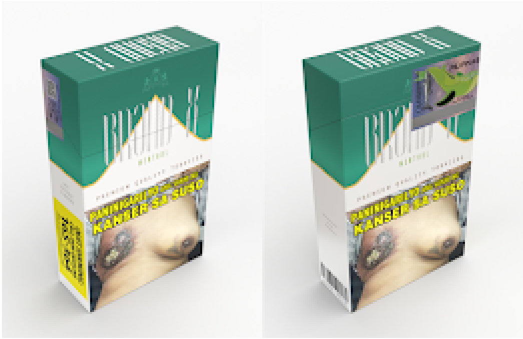

( ) Pack A

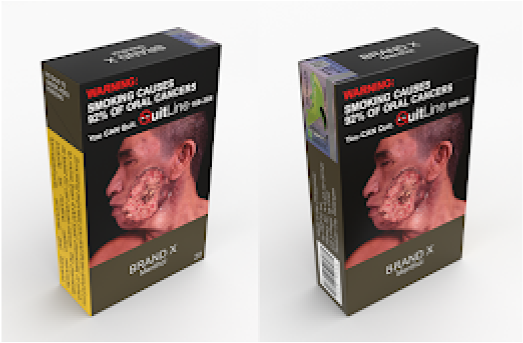

( ) Pack B

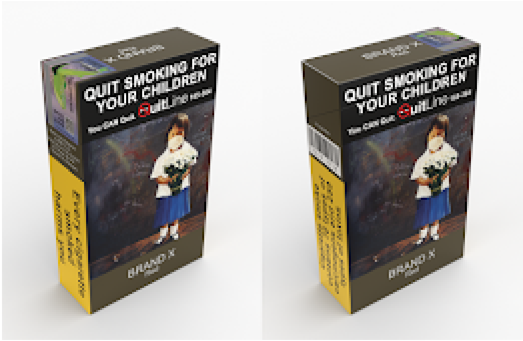

( ) Pack C

Skip to question 30

Any comments or suggestions?

30. Do you have any comments or suggestions to improve this survey?

Thank you very much for your time!

This brings you to the end of the survey. Thank you very much for your time!

Thinking of quitting? For questions about your own smoking habit, you could talk to your doctor
or reach out to the Department of Health’s quit line: 165-364 or SMS 09212039534 or
09776277539.

END OF SURVEY

This content is neither created nor endorsed by Google.

Google Forms
