# Supplementary material for: Graphic health warnings and plain packaging in the Philippines: results of online and household surveys
Source: Front Public Health. 2023 Sep 26;11:1207779. doi: 10.3389/fpubh.2023.1207779 (PMC10562603; doi:10.3389/fpubh.2023.1207779)
Supplement: Supplementary file 3 [file Data_Sheet_2.PDF]

Ateneo School of Government
March 1, 2021

What do Filipinos think of cigarette packaging?

You can help us find out! The Ateneo School of Government-Ateneo Policy Center launches this online survey to study how graphic health warnings on cigarette packs affect smoking behavior among Filipinos and explore the potential of plain packaging of cigarettes in the Philippines. The results will support the Department of Health in shaping policies on tobacco control.

To take part, here's what you need to know:
- It takes about 5 to 10 minutes
- It's completely voluntary
- You don't have to answer any question you don't want to and you can stop at any time
- You and your survey responses will remain anonymous and confidential.

By proceeding to the survey, you voluntarily agree to participate and you're 18 years old and above, and you're not employed or have been employed by the tobacco industry.

Answer the survey through this link:
https://script.google.com/.../AKfycbyYYypKOTeNpfn23j.../exec

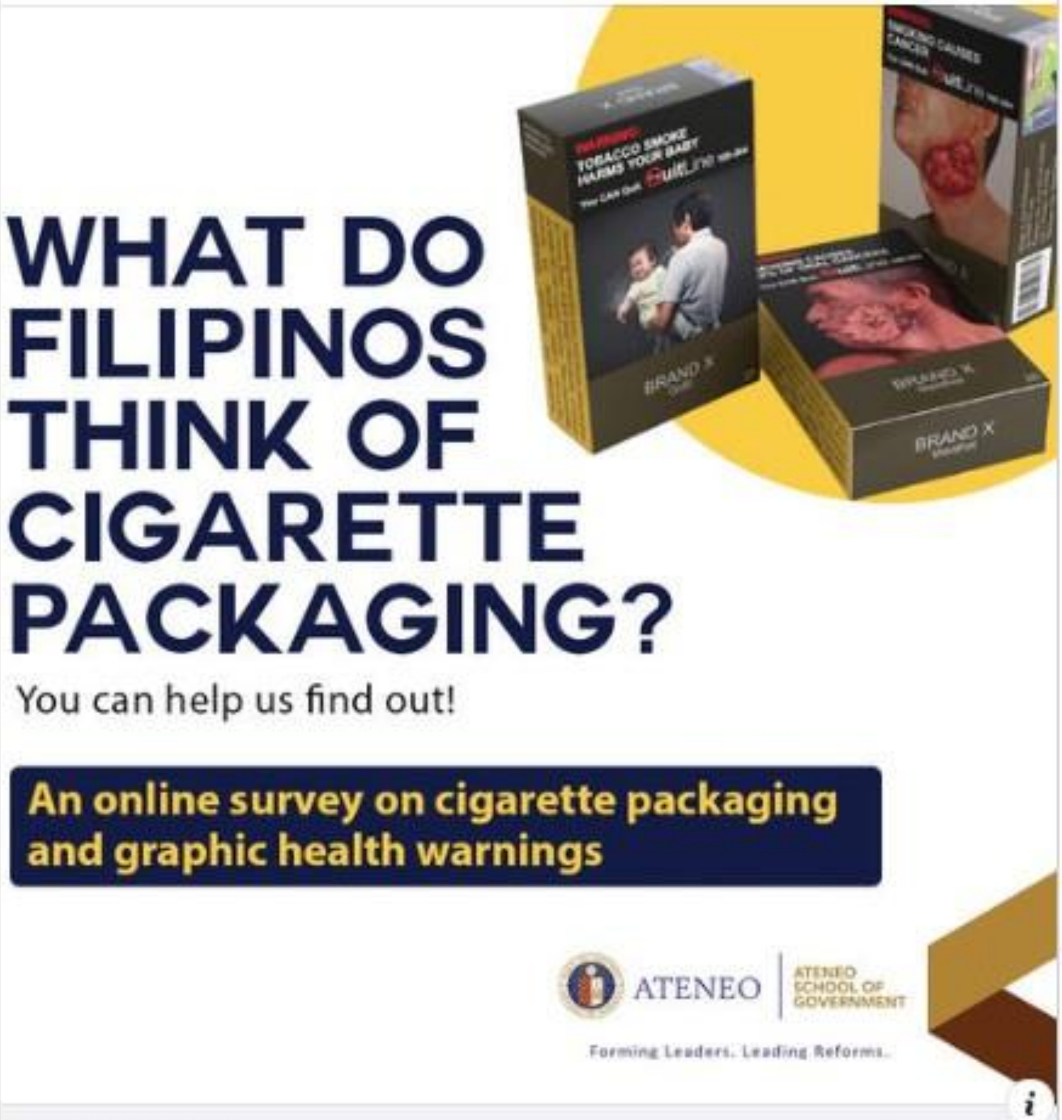

SCRIPT.GOOGLE.COM
script.google.com
Learn more
